# Evolution of Brain-Expressed Biogenic Amine Receptors into Olfactory Trace Amine-Associated Receptors

**DOI:** 10.1093/molbev/msac006

**Published:** 2022-01-11

**Authors:** Lingna Guo, Wenxuan Dai, Zhengrong Xu, Qiaoyi Liang, Eliot T Miller, Shengju Li, Xia Gao, Maude W Baldwin, Renjie Chai, Qian Li

**Affiliations:** 1 Center for Brain Science, Shanghai Children’s Medical Center, School of Medicine, Shanghai Jiao Tong University, Shanghai, China; 2 Department of Anatomy and Physiology, Ministry of Education-Shanghai Key Laboratory of Children's Environmental Health in Xinhua Hospital, Shanghai Jiao Tong University School of Medicine, Shanghai, China; 3 State Key Laboratory of Bioelectronics, Department of Otolaryngology Head and Neck Surgery, Zhongda Hospital, School of Life Sciences and Technology, Jiangsu Province High-Tech Key Laboratory for Bio-Medical Research, Southeast University, Nanjing, China; 4 Co-Innovation Center of Neuroregeneration, Nantong University, Nantong, China; 5 Department of Otolaryngology Head and Neck Surgery, Affiliated Drum Tower Hospital of Nanjing University Medical School, Jiangsu Provincial Key Medical Discipline (Laboratory), Nanjing, China; 6 Research Institute of Otolaryngology, Nanjing, China; 7 Max Planck Institute for Ornithology, Evolution of Sensory Systems Research Group, Seewiesen, Germany; 8 Macaulay Library, Cornell Lab of Ornithology, Ithaca, NY, USA; 9 Shanghai Research Center for Brain Science and Brain-Inspired Intelligence, Shanghai, China

**Keywords:** trace amine-associated receptor, olfactory receptor, GPCR, receptor evolution, receptor deorphanization, site-directed mutagenesis, homology modeling

## Abstract

The family of trace amine-associated receptors (TAARs) is distantly related to G protein-coupled biogenic aminergic receptors. TAARs are found in the brain as well as in the olfactory epithelium where they detect biogenic amines. However, the functional relationship of receptors from distinct TAAR subfamilies and in different species is still uncertain. Here, we perform a thorough phylogenetic analysis of 702 TAAR-like (TARL) and TAAR sequences from 48 species. We show that a clade of *Tarl* genes has greatly expanded in lampreys, whereas the other *Tarl* clade consists of only one or two orthologs in jawed vertebrates and is lost in amniotes. We also identify two small clades of *Taar* genes in sharks related to the remaining *Taar* genes in bony vertebrates, which are divided into four major clades. We further identify ligands for 61 orphan TARLs and TAARs from sea lamprey, shark, ray-finned fishes, and mammals, as well as novel ligands for two 5-hydroxytryptamine receptor 4 orthologs, a serotonin receptor subtype closely related to TAARs. Our results reveal a pattern of functional convergence and segregation: TARLs from sea lamprey and bony vertebrate olfactory TAARs underwent independent expansions to function as chemosensory receptors, whereas TARLs from jawed vertebrates retain ancestral response profiles and may have similar functions to TAAR1 in the brain. Overall, our data provide a comprehensive understanding of the evolution and ligand recognition profiles of TAARs and TARLs.

## Introduction

Trace amine-associated receptors (TAARs) form a distinct subfamily of G protein-coupled receptors (GPCRs) that are specialized to detect both endogenous and exogenous biogenic amines. TAARs were initially discovered as receptors for a number of trace amines that are structurally similar to monoamine neurotransmitters (e.g., serotonin, dopamine, and histamine) but present in trace concentrations in the brain (Borowsky et al. 2001; Bunzow et al. 2001). The subsequent studies revealed a functional dichotomy of TAARs, with TAAR1 expressed in the brain and sensing endogenous trace amines, and all of the other TAARs highly expressed in the main olfactory epithelium (MOE) recognizing exogenous biogenic amines (Liberles and Buck 2006; Xu and Li 2020; Dewan 2021). Several TAARs are also distributed throughout the body and are found in other tissues such as kidney, heart, and testes, albeit with lower expression levels (Gainetdinov et al. 2018). Nonolfactory TAAR1 negatively regulates excitability and monoamine neurotransmitter transmission of dopaminergic and serotonergic neurons. Psychostimulant abuse-related behaviors are reduced by TAAR1 activation and enhanced by TAAR1 knockout in mice (Bradaia et al. 2009; Revel et al. 2011; Achat-Mendes et al. 2012; Liu et al. 2020). Olfactory TAARs, on the other hand, are crucial for perception of amines, an ecologically important class of odorants. Amines are produced by amino acid decarboxylation during decomposition of proteins, and are often enriched in animal body fluids. Therefore, they have been proposed to mediate intra- and interspecific communication through the TAAR olfactory subsystem. For instance, trimethylamine is a species- and sex-specific urine odor that activates TAAR5 to attract mice and repel rats (Li et al. 2013; Saraiva et al. 2016). The TAAR4 ligand 2-phenylethylamine is enriched in predator urine and elicits innate avoidance behaviors in rodents (Ferrero et al. 2011; Dewan et al. 2013). Cadaverine can be produced from decaying animal carcasses and activates TAAR13c, triggering aversive behavior in zebrafish (Hussain et al. 2013). Thus, TAARs play important roles in both psychostimulant addiction and social behaviors.

Since *Taar* genes were first cloned in 2001, they have been identified in several vertebrate genomes (Hussain et al. 2009; Gao et al. 2017; Eyun 2019; Sharma et al. 2019; Dieris et al. 2021). The numbers of intact *Taar* genes vary across species, ranging from zero in dolphins, six in humans, 15 in mice to 112 in zebrafish. TAARs are distantly related to classic biogenic amine receptors, including dopamine and serotonin receptors. Phylogenetic studies have indicated that the 5-hydroxytryptamine receptor 4 (HTR4), a serotonin receptor subtype, is more closely related to the TAAR subfamily than other biogenic amine receptors (Hashiguchi and Nishida 2007; Hussain et al. 2009; Li and Liberles 2016; Dieris et al. 2021). However, there has been controversy over the exact timing of the origin of *Taar* genes. Initial reports suggested that the *Taar* gene family emerged in jawless vertebrates such as sea lamprey (Hashiguchi and Nishida 2007; Libants et al. 2009). Other studies suggested a later origin in early jawed fish (Hussain et al. 2009; Eyun et al. 2016); because so-called sea lamprey TAARs form a monophyletic clade and lack the classic TAAR motif in the transmembrane α-helix VII, they were named *Taar*-like (*Tarl*) genes. The earliest-branching *Taar* genes with the intact TAAR motif are found in cartilaginous fishes, the earliest-branching extant jawed vertebrates, including elephant shark, catshark, white shark, and whale shark (Hussain et al. 2009; Marra et al. 2019; Sharma et al. 2019). Recently, a comprehensive phylogenetic analysis of *Tarl* and *Taar* genes places lamprey *Tarls* as sister to other *Tarl* genes (called the *Taar* V subfamily in previous studies) (Hashiguchi and Nishida 2007; Eyun et al. 2016), which together are sister to the classical *Taars* (Dieris et al. 2021).

Apart from the *Tarl* clade, classical TAAR receptors are classified into three major clades (called class I, II, and III by Hussain et al. 2009, or called clades I, II, and III by Li et al. [2015]). The tetrapod TAARs are grouped in clades I and II, whereas clade III is teleost-specific. Interestingly, almost all of the clade I and II TAARs possess the canonical amine-recognition motif (Asp^3.32^; Ballesteros–Weinstein indexing for GPCRs). This motif is lost in the vast majority of clade III TAARs, which evolve the noncanonical amine-recognition motif, Asp^5.42^. Furthermore, a few TAARs contain both Asp^3.32^ and Asp^5.42^, and recognize diamines (which have two amino groups) (Li et al. 2015). However, with the exception of receptors from a few model species, most of the TAARs in different species remain orphan receptors, which restrict the functional analyses of this specific family of receptors.

Here, we sought to investigate the evolutionary history and functional responses of TAARs, TARLs, and HTR4s. In total, we retrieved 702 TAAR and TARL sequences from 48 vertebrate species and constructed a phylogenetic tree. Among several aminergic receptors selected as outgroups, HTR4s are the closest relative to TAARs and TARLs. TARLs are grouped into two subfamilies. One subfamily expands greatly in lampreys, whereas the other one exhibits very little duplication in jawed vertebrates and appears to be lost in amniotes. We also found that TAARs can be clustered into four distinct clades—Ia, Ib, II, and III. To further analyze their functional relationships, we next identified novel ligands for 61 TAARs and TARLs as well as two HTR4s. Surprisingly, HTR4s, TARLs from jawed fishes and nonolfactory TAAR1 have similar ligand response profiles, whereas some lamprey TARLs recognize ligands that are also recognized by olfactory TAARs, albeit with a distinct structural basis. Consistent with the distinct functional profile of the two TARL subfamilies, we found that TARLs in jawed fishes such as zebrafish are expressed in the brain, whereas lamprey TARLs are expressed in the MOE. In sum, our comprehensive analysis of the evolution and function of TAARs/TARLs uncovers the evolutionary transitions from brain-expressed biogenic amine receptors to olfactory amine detectors. Our study provides evidence for functional distinction and convergence between TAARs and TARLs.

## Results

### Evolutionary History of HTR4s, TARLs, and TAARs

To explore the evolutionary relationships of HTR4s, TARLs, and TAARs, we retrieved a thorough collection of sequences from different species. These species included two jawless fishes (hagfish and sea lamprey), three cartilaginous fishes (ghost shark, bamboo shark, and whale shark), seven teleost fishes (medaka, catfish, goldfish, pufferfish, stickleback, fugu, and zebrafish), one holost fish (spotted gar), one lobe-finned fish (coelacanth), one amphibian (clawed frog), seven reptiles, and 26 mammalian species. In total, 17 sequences of HTR4s, and 702 homologous sequences of TARLs or TAARs from 48 species were obtained (supplementary table S1 and data S1, Supplementary Material online). In addition, we selected 39 classical biogenic amine receptors as outgroups, consisting of seven α adrenergic receptors, three β adrenergic receptors, four dopamine receptors, nine muscarinic acetylcholine receptors, seven histamine receptors, and nine serotonin receptors from whale shark, zebrafish, coelacanth, and mouse.

**Fig. 1.**
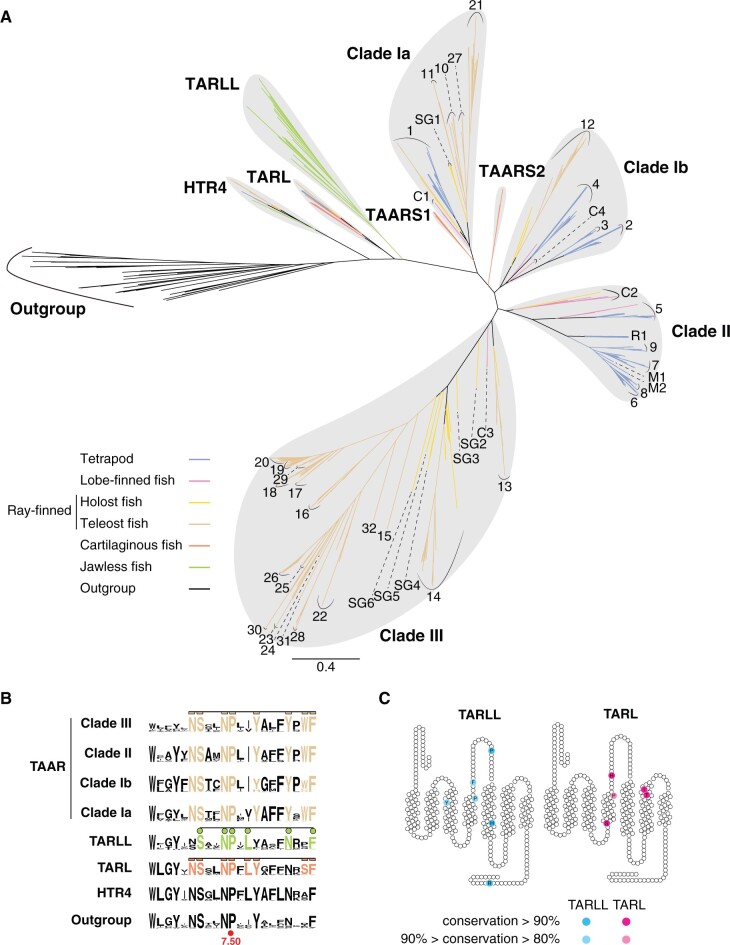
Phylogenetic analysis showing the evolutionary history of HTR4, TAARs and TAAR-like receptors (TARL and TARLL). (*A*) Radial layout of ML phylogenetic tree of HTR4, TAARs, TARLs, and TARLLs. About 758 amino acid sequences from 48 species were included for phylogenetic inference. Outgroups include seven α adrenergic receptors, three β adrenergic receptors, four dopamine receptors, nine muscarinic acetylcholine receptors, seven histamine receptors, and nine serotonin receptors from whale shark, zebrafish, coelacanth, and mouse. Different groups of vertebrate species are color-coded (scale bar=0.4 substitutions per site). According to our updated nomenclature, clade Ia TAARs are mainly composed of TAARC1, TAARSG1, TAAR1 from jawed vertebrates, and teleost-specific TAAR10, 11, 21, 27 subfamilies. Clade Ib TAARs contain TAARC4, tetrapod-specific TAAR2–4, and teleost-specific TAAR12 subfamilies. Clade II TAARs are composed of TAARC2, TAARR1, TAARM1–2, and mammal-specific TAAR5–9 subfamilies. Clade III TAARs contain TAARC3, TAARSG2–6, and teleost-specific TAAR13–20, 22–26, 28–31 subfamilies. (*B*) Sequence logo plots of transmembrane segment VII of the four major TAAR clades (clades Ia, Ib, II, and III), as well as of TARLL, TARL, HTR4, and outgroup receptors. The brown amino acids represent the TAAR fingerprint motif. The green and orange amino acids represent the motifs for TARLL and TARL, respectively. The most conserved proline at 7.50 site in the Ballesteros–Weinstein indexing system is also labeled. (*C*) Conserved sites in TARLL and TARL subfamilies shown in the transmembrane segments and extracellular loops are shown.

The phylogenetic analyses of the receptor sequences were performed using maximal likelihood (ML) in IQ-TREE (supplementary fig. S1*A* and *B*, Supplementary Material online) and nodal support was assessed both by 5,000 ultrafast (UF) bootstrap replicates (Hoang et al. 2018) as well 100 standard bootstrap replicates; similar tree topologies and support values were obtained from multiple independent runs. Consistent with previous results, we recover monophyletic clades of HTR4s, lamprey TARLs, and TAARs with maximal support (fig. 1*A* and supplementary fig. S1*A* and *B*, Supplementary Material online). Like in lamprey TARLs, the classical TAAR motif is also missing in TARLs from this clade (fig. 1*B*). To differentiate between those TARLs from the jawed vertebrates and the lamprey TARLs, we renamed the lamprey TARLs as TARLLs (the last L indicating lamprey). The TARLLs show a large expansion, consisting of 33 members in sea lamprey that can be further subdivided into four subfamilies, TARLL1-4 (supplementary fig. S1*A*, Supplementary Material online). By contrast, there appeared to be generally only one TARL in most of jawed vertebrates (TARL1 in cartilaginous fishes, ray-finned fishes, coelacanth, and amphibians) and two TARLs in some species (TARL1 and TARL2 in whale shark and coelacanth, supplementary fig. S1*A*, Supplementary Material online). We did not find any TARLs in amniotes (supplementary table S1, Supplementary Material online). TARLLs and TARLs therefore display distinct diversification patterns in jawless and jawed vertebrates, suggesting distinct functional roles.

Next, we investigated the evolutionary dynamics of TAARs. TAARs form a large monophyletic clade, including receptors from species ranging from jawed fishes, amphibians, reptiles, and mammals. The TAARs can be further segregated into four major monophyletic groups or clades (Ia, Ib, II, and III), largely corresponding to previously defined groups (Hussain et al. 2009; Li et al. 2015). A previous study investigated around 300 TAAR sequences and segregated them into three clades designated class I, II, and III (Hussain et al. 2009). In our study, with an expanded set of species analyzed, class I TAARs were separated into two clades, which we named clades Ia and Ib. Furthermore, Hussain et al. subdivided TAARs into 28 different subfamilies (TAAR1–28). Here, we found several TAARs that could not be included into the existing 28 subfamilies, so we applied new subfamily names to those TAARs (subfamilies are distinguished if they form separate groups inside the clades). For clade III teleost TAARs, we additionally named three subfamilies (TAAR29, 30, and 31). For other TAARs, we named the subfamilies by the main species in which they occur, including TAARS1–2 (S for shark), TAARC1–3 (C for coelacanth), TAARSG1–6 (SG for spotted gar), TAARR1 (R for reptile), and TAARM1–2 (M for metatherian) (fig. 1*A*). Interestingly, TAARs from cartilaginous fishes display a distinct phylogenetic distribution. Cartilaginous fish TAARs form two subfamilies, TAARS1 and TAARS2. TAARS1 is sister to the remaining clade Ia TAARs, whereas TAARS2 is sister to all subsequent TAARs, including clades Ib, II, and III TAARs (fig. 1*A*). This phylogenetic pattern suggests that the divergence of clade Ia and other TAARs (clades Ib, II, and III) occurred prior to the divergence of cartilaginous fish and bony fish.

The characteristic fingerprint motif (NSxxNPxxYxxxYxWF, where x represents nonconserved amino acids) in the transmembrane α-helix VII was identified for TAARs (Hussain et al. 2009). We analyzed the consensus motifs in TARLLs, TARLs, and the four clades of TAARs (supplementary fig. S2*A*, Supplementary Material online). In agreement with the previous study, the TAAR motif is well conserved in all of the four clades of TAARs. In TARLL and TARLs, we identified similar but distinct fingerprint motifs. TARLLs have a consensus motif of SxxNPxLxxxxNxxF, whereas TARLs have a consensus motif of NSxxNPxLYxxxxxSF (fig. 1*B*). We also identified several conserved amino acids of TARLs and TARLLs that are absent in TAARs. Most of those are in transmembrane or extracellular regions, suggesting a role in ligand recognition, distinct from that seen in TAARs (fig. 1*C*).

We further analyzed the presence of pseudogenes in several species. We did not find any TAAR/TARL/TARLL pseudogenes in hagfish, confirming that TARLLs specifically emerge in lamprey. The percentages of pseudogenes differ across species, varying from 6% in mouse to 44% in whale shark (supplementary fig. S2*B*, Supplementary Material online).

### Cell-Based High-Throughput Screening to Deorphanize Receptors

**Fig. 2.**
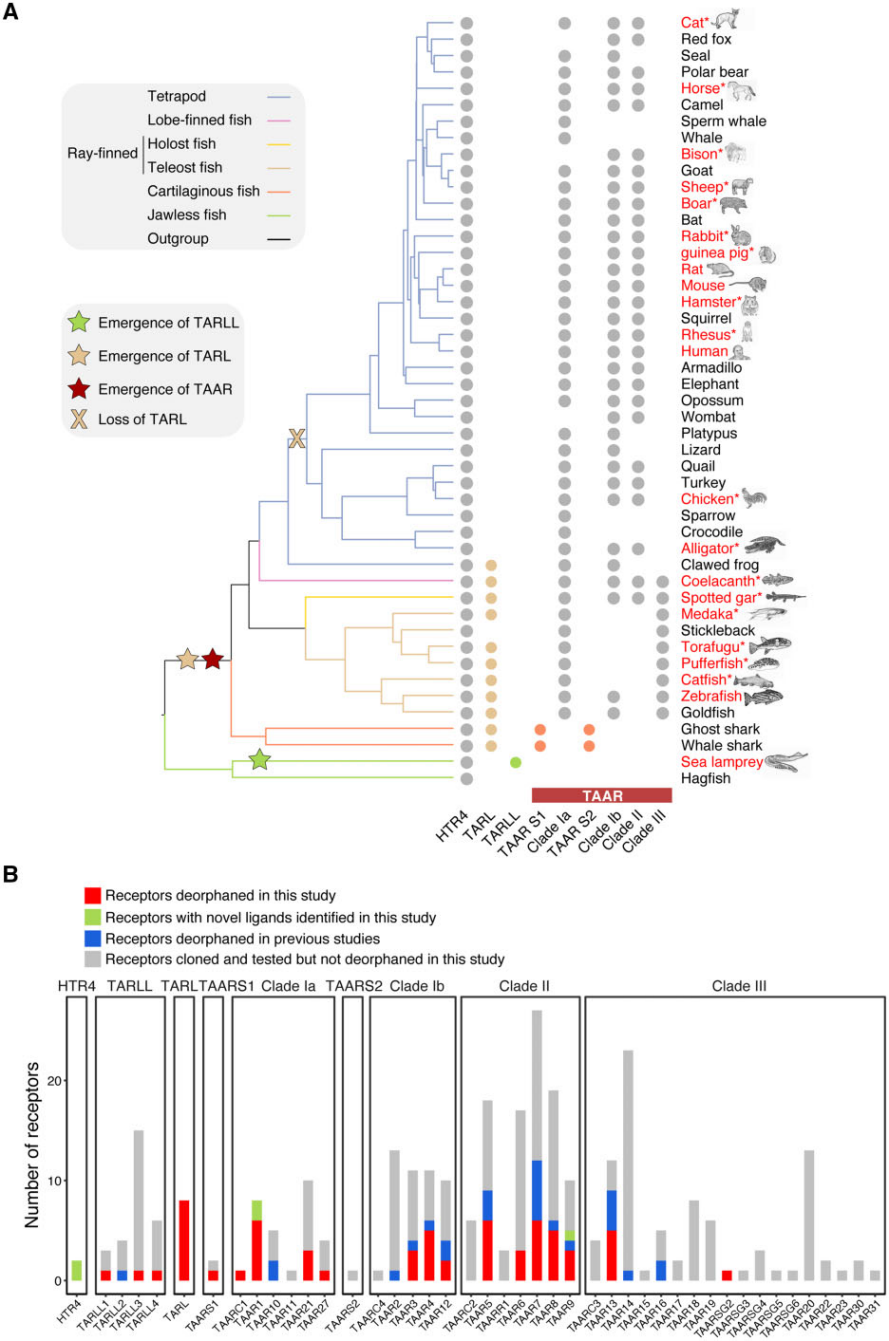
Summary of deorphaned receptors. (*A*) The evolutionary relationship of 47 of the 48 species (except bamboo shark) whose receptors were included in this study were depicted. The presence of receptor genes was marked by the filled circles, which are color-coded according to phylogenetic group as in figure 1*A*. The emergence and loss of TARLL, TARL, and TAAR clades are indicated by colored stars and cross. Names of species in red text indicate a species whose receptors were cloned and tested in this study. The asterisks signify that ligands for TAARs of those species were not studied before. (*B*) The numbers of receptors cloned and deorphaned were summarized, and divided into four groups: receptors newly deorphaned in this study (red), receptors with novel ligands identified in this study (light green), receptors deorphaned in previous studies (light blue), and receptors cloned and tested but not deorphaned (gray).

Next, we conducted high-throughput screening to further explore the functional properties of these aminergic receptors. We cloned 248 TAARs from 11 mammals (human, mouse, rat, guinea pig, hamster, cat, horse, rabbit, sheep, rhesus, and wild boar), two reptiles (alligator and chicken), six teleosts (zebrafish, catfish, fugu, medaka, salmon, and pufferfish), one lobed-finned fish (coelacanth), one holost fish (spotted gar), and one cartilaginous fish (bamboo shark) (fig. 2*A*). We also cloned eight TARLs from coelacanth, zebrafish, catfish, fugu, medaka, spotted gar, shark, and 28 TARLLs from sea lamprey (supplementary table S2, Supplementary Material online). In addition, we cloned two HTR4s from mouse and zebrafish. We then challenged all of the cloned receptors with a ligand library comprised 97 amines at 100 µM. In total, we successfully identified novel ligands for 50 TAARs, eight TARLs, three TARLLs, and two HTR4s. The deorphaned receptors are spread across the major receptor clades (fig. 2*B* and supplementary table S3, Supplementary Material online). For those receptors showing high responses, we subsequently performed dose–response curves. Most of the ligands activate the receptors with half maximal effective concentrations (EC_50_) of 1–100 µM, and a few ligands show extremely sensitive activation with an EC_50_ of 20 pM (supplementary table S4, Supplementary Material online).

To better understand the ligand response profiles, we classified the 97 amines into eight chemical clusters according to their chemical features, including predefined atom symbols, bond types, atom environment properties, and atom properties (supplementary fig. S3, Supplementary Material online). The largest three clusters consist of a group mainly composed of primary or secondary amines (cluster 2), another group is primarily composed of amines with aromatic rings (cluster 3), and cluster 5 is mainly composed of tertiary amines. The other five clusters include cluster 1 (taurine), cluster 4 (creatinine), cluster 6 (isopropylamine), cluster 7 (aniline) and cluster 8 (indole and its derivatives). It is worth noting that most biogenic amine neurotransmitters are included in chemical cluster 3, and most of the other known TAAR ligands are included in clusters 2 and 5. The 52 identified ligands are mainly in cluster 2, 3, 5, 6, 7, and 8. All of the molecules are positively charged in physiological conditions, except for molecules in cluster 7 and cluster 8, which are neutral in physiological conditions.

### Functional Relationships of HTR4s, TARLLs, TARLs, and TAARs

**Fig. 3.**
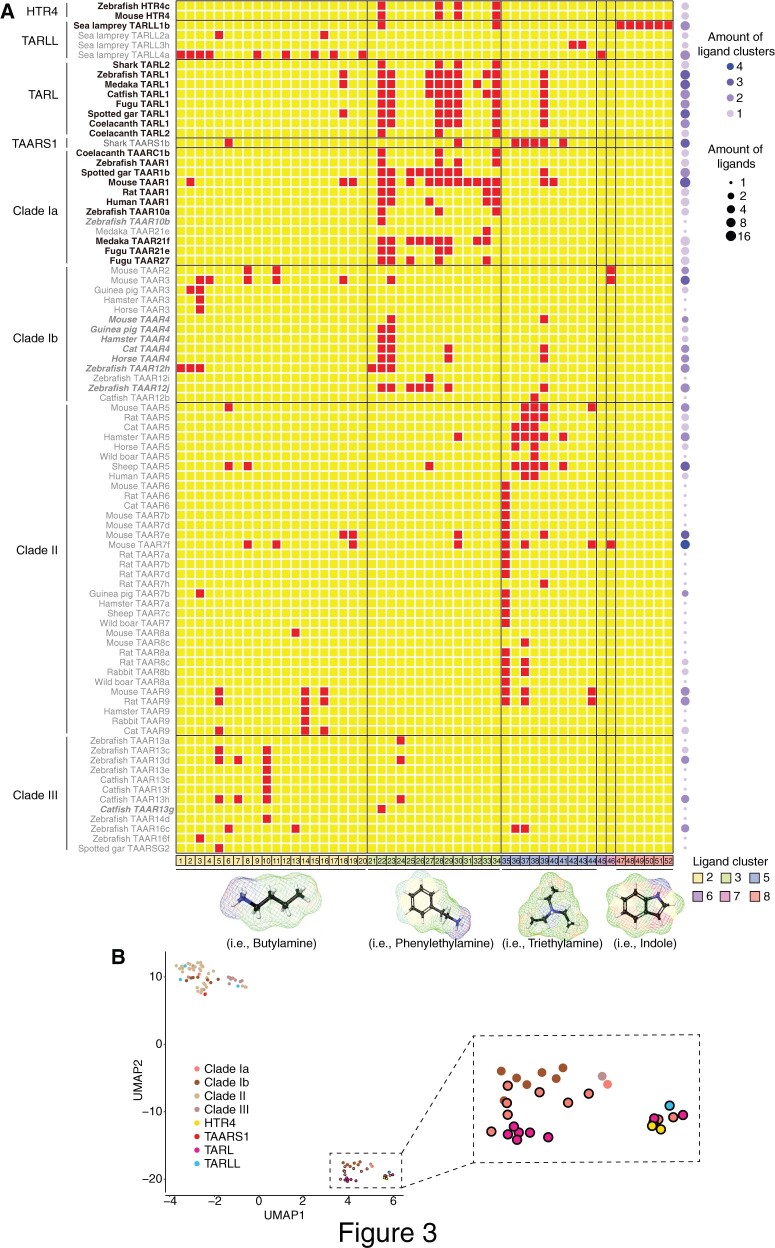
Ligand response profile of HTR4s, TARLs, TARLLs, and TAARs. (*A*) Overview of the ligand response profile of all of the deorphaned receptors. The red squares represent positive ligand–receptor interaction and the yellow squares denote no responses. The horizontal lines show borders of different clades. The tested compounds were classified into eight chemical clusters (supplementary fig. S4, Supplementary Material online) (only six clusters of compounds were found to be ligands of any receptor); different clusters are separated by the vertical lines. The molecule structures underneath panel (*A*) are chosen as representatives of each chemical cluster. The names of those ligands are shown below: 1) butylamine, 2) hexylamine, 3) isoamylamine, 4) isobutylamine, 5) cadaverine, 6) pyrrolidine, 7) putrescine, 8) 2-methylbutylamine, 9) 3-methylthiopropylamine, 10) agmatine, 11) cyclohexylamine, 12) cyclopentylamine, 13) piperidine, 14) spermidine, 15) 5-amino-1-pentanol, 16) spermine, 17) amylamine, 18) octylamine, 19) heptylamine, 20) propylamine, 21) benzylamine, 22) tryptamine, 23) phenylethylamine, 24) histamine, 25) tyramine, 26) octopamine, 27) 3-methoxytyramine, 28) 5-methoxytryptamine, 29) 4-methoxyphenethylamine, 30) 5-methoxy-*N*,*N*-dimethyltryptamine, 31) gramine, 32) phenylethanolamine, 33) dopamine, 34) serotonin, 35) *N*,*N*-dimethylcyclohexylamine, 36) 1-methylpyrrolidine, 37) 1-methylpiperidine, 38) trimethylamine, 39) *N*,*N*-dimethylphenethylamine, 40) *N*,*N*-dimethyl benzyl amine, 41) *N*,*N*-dimethylisopropylamine, 42) *N*,*N*,*N*′,*N*′-tetramethyl-1,4-butanediamine, 43) *N*,*N*,*N*′,*N*′-Tetramethyl-1,3-propanediamine, 44) triethylamine, 45) isopropylamine, 46) aniline, 47) indole, 48) 1-methylindole, 49) 3-methylindole, 50) 5-methylindole, 51) 6-methylindole, and 52) 7-methylindole. The tuning breadth of each receptor is color-coded on the right. (*B*) The UMAP reduction and visualization of phyloPCA results of the deorphaned receptors is shown. The cluster in the lower right quadrant is outlined with a dashed rectangle and highlighted in the inset. Within this cluster, dots with black outlines indicate the receptors (HTR4, TARL, clade Ia TAAR, and a TARLL) which belong to group 2 by the PAM clustering method; dots are colored according to phylogenetic clade. Note that the receptor names of this group (PAM group 2) are colored in black in (*A*); the names of PAM group 1 receptors close to PAM group 2 are italicized in (*A*); all other names of PAM group 1 receptors are in gray.

Next, we combined our data with known ligands of TAARs from model organisms, such as human, mouse, rat, and zebrafish (Xu and Li 2020). Comparison of ligand responses showed that in general, receptors in different clades have distinct ligand profiles, responding to chemicals in distinct clusters, whereas receptors within the same clades tend to detect ligands from the same chemical clusters (fig. 3*A*). Further, TAAR and TARL orthologs from different species generally detect very similar ligands, suggesting conservation of receptor function across species (fig. 3*A*). However, we did observe broader ligand profiles for receptor orthologs in some species, although we could not rule out the possibility that receptor orthologs may not function equally well in vitro. It is also worth noting that TAAR-expressing olfactory sensory neurons generally recognize the same ligands identified by in vitro assays, yet with enhanced sensitivity and increased number of ligands, likely due to optimized signaling components and chaperons in neurons in vivo (Pacifico et al. 2012; Zhang et al. 2013).

To better illustrate the functional relevance of HTR4s, TARLLs, TARLs, and TAARs, we performed phylogenetic principal component analysis (phyloPCA) (Revell and Collar 2009) on all of the deorphaned receptors based on the ligand profiles and the ML phylogenetic tree. In our primary data set, since the top six principal components (PCs) together only explain 63.9% of the variance (supplementary fig. S4*A–C*, Supplementary Material online), we further applied dimensionality reduction using Uniform Manifold Approximation and Projection (UMAP) on all phyloPCA scores for better visualization (fig. 3*B*). To quantitatively classify receptors by their phyloPCA scores on all PC axes, we used a clustering algorithm (Partition Around Medoids, PAM) after identifying the optimal number of groups (Maechler et al. 2019; Kassambara and Mundt 2020). We found that all of these receptors were assigned into two groups by PAM (supplementary fig. S4*D*, Supplementary Material online), overlapping with the UMAP visualization (fig. 3*B*). We noted that almost all of the receptors from clade Ia TAARs (except zebrafish TAAR10b and medaka TAAR21e), TARLs, and HTR4s are clustered in PAM group 2, suggesting that they share similar ligand response profiles. Some clade Ib TAARs (TAAR4 and TAAR12) and a clade III TAAR (catfish TAAR13g) also appear in the UMAP plot close to PAM group 2, which might be due to their responses to some ligands in cluster 3 (amines with aromatic rings). However, most clades Ib, II, and III TAARs cluster in PAM group 1 together with shark TAARS1b both in the PAM clustering and the UMAP plot. For the four deorphaned sea lamprey TARLLs, three (TARLL2a, TARLL3h, and TARLL4a) are clustered into PAM group 1, and only TARLL1b is clustered into PAM group 2. Since Dieris et al. (2021) showed a different topology on the clades of TARLL, TARL, and TAAR with higher support values, we also examined the effect of the difference in topologies by analyzing responses of a reduced set of receptors present in both phylogenies. The clustering results from the topology comparison are similar, and are consistent with the results from the full data set (supplementary fig. S5*A–D*, Supplementary Material online).

### TAARs Show Distinct Ligand-Binding Features

We identified novel ligands for one TAARS1 (TAARS1b), five clade Ia, ten clade Ib, 24 clade II, and ten clade III TAARs from 18 different species (supplementary table S3, Supplementary Material online), providing an opportunity to understand TAAR-ligand interaction in depth. We previously reported that clade III TAARs evolved the noncanonical Asp^5.42^ to detect amine ligands, whereas other TAARs utilize the conserved Asp^3.32^ to detect amine ligands (Li et al. 2015). We observed the same pattern in the current study (supplementary fig. S6*A*, Supplementary Material online). Further, we found that Asp^3.32^ (but not Asp^5.42^) is well conserved in sequences of HTR4, TARLL, and TARL, implying that HTR4, TARLL, and TARL use the conserved Asp^3.32^ to detect amines. Interestingly, a few clade II mammalian TAARs, including TAAR6 and TAAR8, have Asp^5.43^ instead of Asp^5.42^. This results from the loss of an amino acid at 5.44 in the fifth transmembrane segments of those receptors (supplementary fig. S6*A*, Supplementary Material online). In spite of this fact, simulations do suggest that those TAARs may recognize diamines according to computational simulations (Izquierdo et al. 2018). However, our results clearly showed that Asp^5.43^-containing TAAR6 and TAAR8 family members, including TAAR6 from mouse, rat, cat, TAAR8a from rat, wild boar, and TAAR8b from rabbit, can only be activated by monoamines other than diamines (supplementary fig. S6*B* and table S3, Supplementary Material online). We suspect that Asp^5.43^ might be directed away from the potential binding pocket, and thus may not be involved in amine recognition.

Our previous study also proposed a two-step model for Asp^5.42^ acquisition, suggesting that an ancestral TAAR of clade III TAARs acquired Asp^5.42^, gaining diamine sensitivity, and subsequently lost Asp^3.32^ (Li et al. 2015). Interestingly, we found that both Asp^3.32^ and Asp^5.42^ are present in the cartilaginous fish TAARS2 subfamily (supplementary fig. S6*A*, Supplementary Material online). The facts that TAARS2 already has both Asp^3.32^ and Asp^5.42^ together with the observation that some clade II mammalian TAARs contain both Asp^3.32^ and Asp^5.43^ suggest two alternative scenarios. In one scenario, these two residues may have arisen independently and convergently multiple times, in shark TAARS2, and in some clade II and clade III TAARs. Alternatively, after the divergence of clade Ia, the ancestor of the subsequent TAARs may have evolved both Asp^3.32^ and Asp^5.42^. Asp^5.42^ may have then been lost in clade Ib TAARs but retained in the common ancestor of clade II and III TAARs, followed by subsequent loss of Asp^5.42^ in some clade II TAARs or shifting to Asp^5.43^ in other clade II TAARs (as a result of a deletion, supplementary fig. S6*A*, Supplementary Material online), and loss of Asp^3.32^ in the majority of clade III TAARs (supplementary fig. S6*C*, Supplementary Material online).

In addition, it was previously reported that clade Ib TAAR2-4 mainly recognize primary and secondary amines, but clade II TAAR5-9 mainly recognize tertiary amines (Ferrero et al. 2012). Here, we found that clade II TAARs can also recognize a relatively large number of cluster 2 ligands with primary and secondary amines. This is consistent with previous studies showing that TAAR3 and TAAR4 are more broadly tuned in vivo, and could recognize tertiary amines as well (Zhang et al. 2013; Dewan et al. 2018). We also found that clade Ib TAARs respond predominantly to cluster 3 ligands (amines with aromatic rings), and clade II TAARs respond predominantly to cluster 5 ligands (tertiary amines) (supplementary fig. S7*A* and *B*, Supplementary Material online).

Another interesting finding is the functional convergence between shark TAARS1 and TAAR5. Shark TAARS1 appears closely related to clade Ia TAARs according to the phylogenetic tree (fig. 1*A*). However, the ligand profile of TAARS1 is more similar to TAAR5 in clade II than to TAARs in clade Ia, which mainly recognizes cluster 3 molecules (amines with aromatic rings). Bamboo shark TAARS1b can recognize one ligand from cluster 2 (primary or secondary amines), one ligand from cluster 3 (amines with aromatic rings), and five ligands from cluster 5 (tertiary amines), which are the ligands of TAAR5 as well (fig. 3*A* and *B*; supplementary fig. S8*A*, Supplementary Material online). We used the R package l1ou (Khabbazian et al. 2016) to search across the phylogenetic tree for well-supported shifts to new optima in ligand response profiles (supplementary fig. S8*B*, Supplementary Material online). A notable shift was indeed discovered in shark TAARS1b following the divergence from clade Ia TAARS (supplementary fig. S8*B*, Supplementary Material online). This functional convergence of TAARS1 and TAAR5 is intriguing: as TAAR5 ligands are potent allomones in mammals (Li et al. 2013), the ecological relevance of TAARS1b ligands for cartilaginous fishes will be interesting to explore.

### Independent Development and Functional Convergence of TARLL and TAAR

**Fig. 4.**
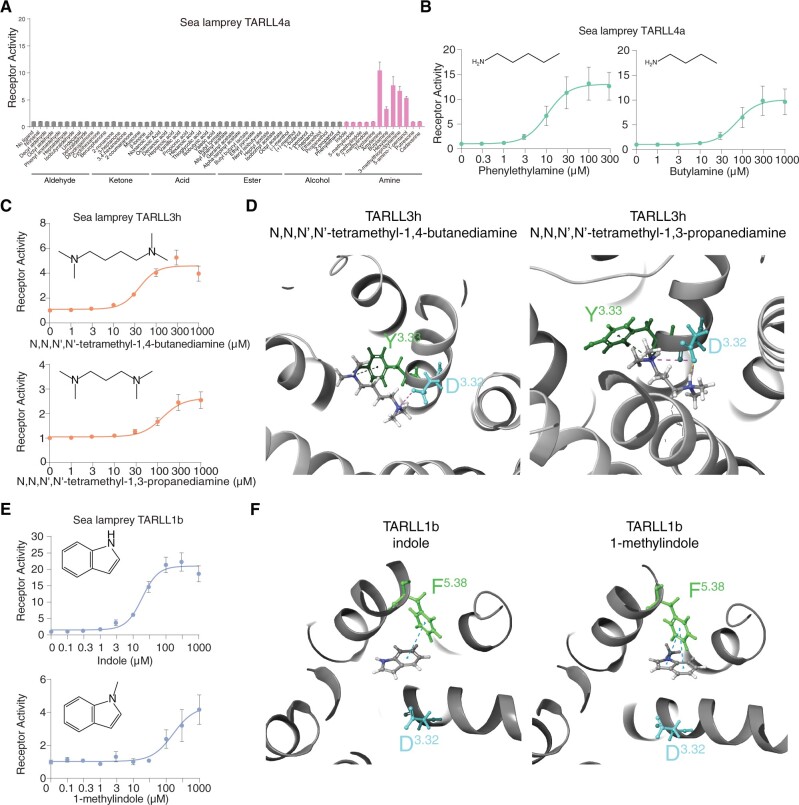
Structural basis for ligand recognition of TARLLs. (*A*) Responses of sea lamprey TARLL4a to different groups of chemicals including aldehyde, ketone, acid, ester, alcohol, and amines (pink color). (*B*) Dose-dependent responses of TARLL4a to phenylethylamine and butylamine. (*C*) Dose-dependent responses of TARLL3h to *N*,*N*,*N*′,*N*′-tetramethyl-1,4-butanediamine and *N*,*N*,*N*′,*N*′-tetramethyl-1,3-propanediamine. (*D*) Homology modeling and ligand docking for TARLL3h binding to two ligands, *N*,*N*,*N*′,*N*′-tetramethyl-1,4-butanediamine (left) and *N*,*N*,*N*′,*N*′-tetramethyl-1,3-propanediamine (right). Both Asp^3.32^ and Tyr^3.33^ were found to interact with ligands. (*E*) Dose-dependent responses of TARLL1b to indole and 1-methylindole. (*F*) Homology modeling and ligand docking for TARLL3h binding to indole and 1-methylindole. Phe^5.38^ was found to play a role in ring recognition of indole through pi–pi interactions.

Next, we examined the TARLL clade in more detail. Previous studies have suggested that TARLLs are expressed in the MOE and mediate olfactory function in the sea lamprey (Scott et al. 2019). However, detailed analyses of those receptors are still lacking. Here, we successfully deorphaned three lamprey TARLLs (TARLL1b, 3 h, 4a) out of 28 cloned and functionally tested receptors, and further investigated the structural basis in TARLLs for ligand recognition (supplementary table S3, Supplementary Material online). Expression patterns of the three related *Tarll* genes determined by in situ hybridization in the MOE of sea lamprey also supports the hypothesis that lamprey TARLLs mainly function as olfactory receptors (supplementary fig. S9*A*, Supplementary Material online). TARLL4a has a broad ligand profile of amines with eight ligands from cluster 2 (primary or secondary amines) and one ligand from cluster 6 (isopropylamine). The responses of TARLLs to amines are very specific as they are not responsive to other chemicals including aldehydes, ketones, acids, esters, and alcohols (fig. 4*A*). The ligand profile includes long alkyl chain amines, such as hexylamine and heptylamine, and amines whose nitrogen is connected to a secondary carbon, such as isopropylamine and cyclohexylamine (fig. 4*B* and supplementary fig. S9*B*, Supplementary Material online). This suggests that the binding pocket of TARLL4a is flexible in its volume to fit the sizes of a variety of ligands.

Interestingly, TARLL3h recognizes tetramethyl-1,4-butanediamine and *N*,*N*,*N*′,*N*′-tetramethyl-1,3-propanediamine, diamines with two amino groups (fig. 4*C*). Recognition of diamines in TAARs involves two aspartic acids, Asp^3.32^ and Asp^5.42^ (Li et al. 2015; Sharma et al. 2016). However, although Asp^3.32^ is retained in TARLL3h, Asp^5.42^ is not found, implying a distinct diamine recognition mechanism in TARLL3h. To investigate this further, we performed homology modeling and ligand docking in TARLL3h. In addition to Asp^3.32^ that forms a salt bridge with *N*,*N*,*N*′,*N*′-tetramethyl-1,4-butanediamine or *N*,*N*,*N*′,*N*′-tetramethyl-1,3-propanediamine, Tyr^3.33^ binds to the other amino group through pi–cation interactions in TARLL3h (fig. 4*D*). Notably, the Tyr^3.33^ site is one of the well-conserved amino acids in the TARLL clade (fig. 1*C*). To verify the docking results, we performed site-directed mutagenesis in TARLL3h. Mutation of Asp^3.32^ or Tyr^3.33^ to alanine (D3.32A or Y3.33A) completely eliminated the receptor activity, whereas mutation of Tyr^3.33^ to phenylalanine (Y3.33F) retained pi–cation interactions and the receptor activity yet resulted in slightly reduced affinity (EC_50_ three times higher than wild type receptor) (supplementary fig. S9*C*, Supplementary Material online). These results suggest the potential roles of Asp^3.32^ and Tyr^3.33^ of TARLL3h in diamine recognition.

Moreover, TARLLs seem to extend ligand profiles beyond the profiles seen in TAARs. TARLL1b can be activated by indole and its derivatives, which are not ligands for any TAARs (fig. 4*E* and supplementary fig. S9*D* and *E*, Supplementary Material online). Indole and its derivatives are neutral in physiological conditions, and are found to activate members of odorant receptor family (Pfister et al. 2020). To probe the structural basis of indole recognition, we performed homology modeling and ligand docking in TARLL1b (fig. 4*F*). The docking results suggested that Phe^5.38^ is the critical site to stabilize indole through pi–pi interactions with the aromatic rings of indole. This site is also important for recognition of indole derivatives such as 1-methylindole (fig. 4*F*).

### TARL and TAAR1 Preserve the Ligand Profiles of HTR4 and Independently Gain New Functions

**Fig. 5.**
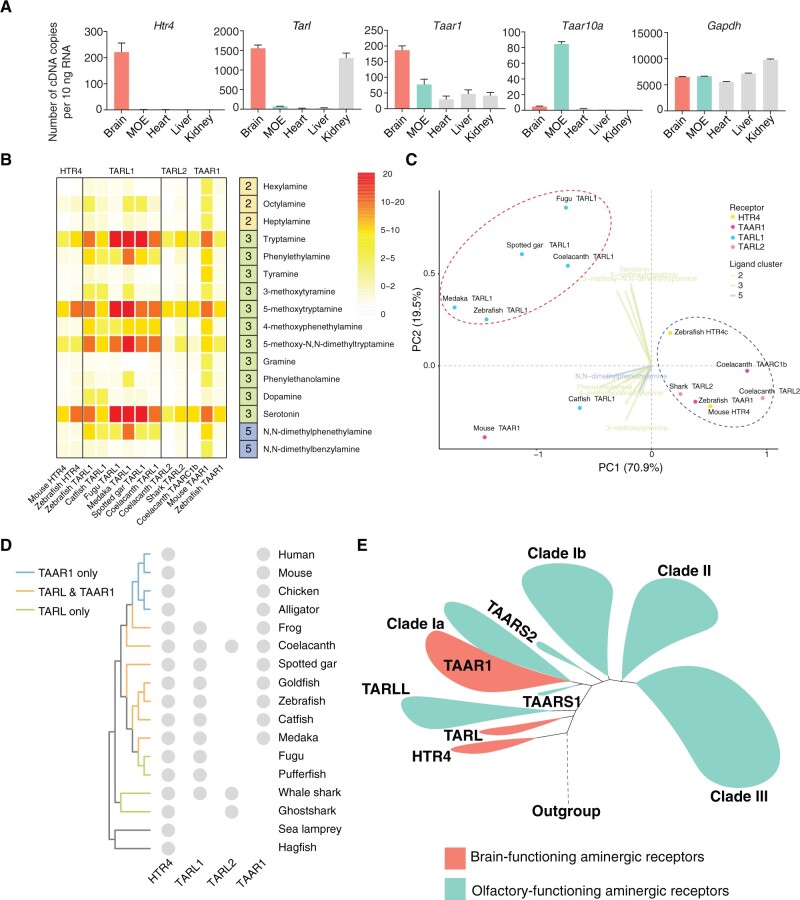
Ligand profiles and expression patterns of HTR4, TARL, and TAAR1. (*A*) qPCR analysis of different receptors in zebrafish tissues. *Gapdh* serves as a control. (*B*) Response of HTR4, TARL, TAARC1, and TAAR1 to 16 ligands at 10 μM. The heatmap is based on the ratio of fluorescence intensity of ligand–receptor interaction and no ligand controls in the luciferase assay. TAARC1 is also included because it resides in clade Ia close to TAAR1 and shows a similar ligand profile to HTR4, TARL, and TAAR. (*C*) PhyloPCA analysis on the responses of HTR4, TARL, TAARC1, and TAAR1 to these 16 ligands. In this analysis, PC1 explains 70.9% of the variance and PC2 explains 19.5% of the variance. Dots colored by receptor clades represent the ligand responses of receptors on the first two PC axes. Ligand eigenvectors are represented by lines and colored according to the ligand clusters. A ligand name is shown at the tip of the line if its eigenvector has a higher contribution (compared with other ligands) to either PC1 or PC2. Red and blue dashed circles indicate two groups of receptors separated by PC1 and PC2. (*D*) The presence of HTR4, TARL1, TARL2, and TAAR1 in several representative species in vertebrates is plotted. (*E*) A model for the evolution and functional diversification of aminergic receptors. The clades labeled in green (including TARLL, TAARS1, TAARS2, the non-TAAR1 part of clade Ia, clade Ib, clade II, and clade III TAARs) play important roles in olfaction. The clades labeled in red, including HTR4, TARL, and TAAR1 are likely to detect amine neurotransmitters and trace amines in the brain.

As underscored by the UMAP plot (fig. 3*B*), TARL, HTR4, and TAAR1 appear to have similar ligand profiles and are cluster together (PAM group 2). Most of their ligands belong to cluster 3 ligands (amines with aromatic rings), which are mainly neurotransmitters and their derivatives (fig. 3*A*). In addition, the analysis of expression levels in a panel of zebrafish tissues demonstrates that these receptors are mainly expressed in the brain, instead of in the MOE where other TAARs and TARLLs are expressed (Liberles and Buck 2006; Scott et al. 2019) (fig. 5*A*). The expression pattern and ligand profile of TARLs suggest a possible function as a neurotransmitter receptor in the brain rather than as peripheral chemosensors.

To better compare their ligand profiles, we selected the 16 ligands that activate HTR4, TARL, and TAAR1 in our screening assay (supplementary table S3, Supplementary Material online). This set included three molecules from chemical cluster 2 (primary or secondary amines), 11 molecules from chemical cluster 3 (amines with aromatic rings), and two molecules from chemical cluster 5 (tertiary amines). We then compared the responses of HTR4 from mouse and zebrafish, TARL1 from zebrafish, catfish, fugu, medaka, spotted gar, and coelacanth, TARL2 from coelacanth and shark, TAAR1 from mouse and zebrafish to the 16 ligands at 10 µM (fig. 5*B*). In general, all receptors showed high activation levels to the four HTR4 ligands, suggesting functional conservation with HTR4. Furthermore, they also displayed distinct ligand profiles. TARL1s seem to have broader ligand profiles compared with HTR4. The TARL1 ligands include not only tryptamine and its derivatives, but also phenylethylamine and its derivatives, which are also brain neurotransmitters but have a totally different aromatic ring structure from tryptamine. Therefore, TARL1 appears not only to preserve the possible ancestral function shared with HTR4, but also gains new functions. In contrast, TARL2s cannot recognize phenylethylamine and its derivatives and have a similar ligand profile to HTR4. Consistent with these results, the phyloPCA analyses showed that TARL1s are clearly separated from HTR4s and TARL2s (fig. 5*C*), and a functional shift was also observed in TARL1s after divergence from TARL2s (supplementary figs. S8*B* and S10*A* and *B*, Supplementary Material online). TAAR1s share a similar ligand profile to HTR4 and TARL as a whole, but also differ across species. Mouse TAAR1 not only recognizes the ligands of HTR4 and TARL, but also has slight responses to long-chain primary monoamines from chemical cluster 2 (hexylamine, heptylamine, and octylamine). Zebrafish TAAR1 and coelacanth TAARC1b, the counterpart of TAAR1 in coelacanth, however, have the same ligand profile as HTR4 (fig. 5*B*). In addition, we tested dose-dependent responses of these receptors to tryptamine and serotonin, and found that the sensitivities vary for different receptors to the same ligand (supplementary fig. S11*A* and *B*, Supplementary Material online).

In summary, we trace the evolution and functional differentiation of the TAAR/TARL family. HTR4 is present in jawless fishes and is retained in all vertebrates. TARL1 exists in cartilaginous fishes, teleost, holost, coelacanth, and in amphibians, but is lost in amniotes. TARL2 emerges in cartilaginous fishes, and is lost in ray-finned fishes and tetrapods (but is present in coelacanth). On the other hand, TAAR1 is found in some ray-finned fish species and is preserved in tetrapod (fig. 5*D*). This phylogenetic distribution, together with the common expression in the brain and the functional conservation suggest that TARL and TAAR1 may have gradually developed new functions in the brain after diverging from a common ancestor shared with HTR4.

## Discussion

In this study, we systematically analyzed the evolutionary history and functional response properties of TAARs. By constructing a phylogeny of 702 TAAR and TARL sequences from 48 vertebrate species, we demonstrated that TAARs and TARLs are very likely derived from an ancestral *Htr4* gene duplication. Furthermore, we found that TARLs form two monophyletic clades: one is lamprey-specific (we named TARLL) and the other includes TARLs from jawed vertebrates. For TAARs, our study classified them into four distinct clades—Ia, Ib, II, and III. Further ligand screening data showed that HTR4s, TARLs from jawed fishes, and the nonolfactory TAAR1 are expressed in the brain and have similar broad ligand profiles. In contrast, lamprey TARLLs recognize distinct ligands that are also recognized by olfactory TAARs, consistent with their specific expression in the MOE. Taken together, our study outlines the evolutionary history and functional evolution of brain-expressed biogenic amine receptors into olfactory amine-detecting receptors, and suggests widespread functional diversification and convergence in TAARs and TARLs.

### Evolution of Brain-Expressed Biogenic Amine Receptors into Olfactory Amine Detectors

Investigating the gain and loss of genes together with shifts in ligand profiles provides important insights into the origin and function of TAARs. Previous evolutionary studies were performed mainly on limited number of TAARs and TARLLs with very few functional analyses (Libants et al. 2009; Hussain et al. 2013; Li et al. 2015; Scott et al. 2019; Dieris et al. 2021). In our study, we performed a thorough phylogenetic analysis of 702 TAAR and TARL sequences from 48 species including jawless fishes, cartilaginous fishes, teleost fishes, holost fishes, lobe-finned fishes, amphibians, reptiles, and mammals. We further cloned and tested 286 receptors, and identified novel ligands for 61 receptors, and integrated the ligand profiles with phylogenetic analyses. Hence, our data provide the most comprehensive evolutionary and functional understanding of TAARs and TARLs to date. Furthermore, we performed sequence analysis and receptor deorphanization in several species including bamboo shark, spotted gar, and coelacanth that have not been studied before. These species are situated at important phylogenetic junctures and provide insight into the functional evolution of this clade of receptors.

Based on the results of phylogenetic analyses and functional assays, we classified the deorphaned receptors into two functional types (fig. 3*B*). One functional group is mainly composed of HTR4s, TARLs, and TAAR1. This group of receptors mainly recognizes amines from chemical cluster 3 (amines with aromatic rings) that are mostly neurotransmitters and their derivatives. The other group of receptors is composed of TARLLs, non-TAAR1 clade Ia TAARs, clade Ib, II, and III TAARs. The ligand profile of this group of receptors is broad, including cluster 2 (primary or secondary amines) and cluster 5 (tertiary amines) ligands that are mostly alkyl amines and are known olfactory odorants. Moreover, the above observations agree with the receptor expression patterns. HTR4s, TARLs, and TAAR1 are primarily expressed in the brain, whereas TARLLs and other TAARs are specifically expressed in the MOE. Therefore, we hypothesize that TARLLs independently expanded in sea lamprey and evolved into olfactory receptors detecting water-soluble amines. By contrast, TARLs from jawed vertebrates are strongly conserved in number and their function was eventually replaced by TAAR1 in amniotes. TAAR1s are nonolfactory, brain-expressed and play an important role in regulating psychostimulant abuse-related behaviors (Bradaia et al. 2009; Revel et al. 2011; Achat-Mendes et al. 2012; Liu et al. 2020), and are proposed to regulate synaptic strength in different brain regions. It is possible that TARLs may function similarly in the brain of jawed vertebrates. As a side note, the distinct expression pattern of TAAR1 in the brain rather than in the MOE could be due to the insulated TAAR1 genomic domain from two newly identified olfactory TAAR enhancers (Fei et al. 2021; Shah et al. 2021).

Given our results, we propose that these receptors may be functionally assigned to two distinct classes: brain-expressed biogenic amine receptors and MOE-expressed olfactory amine detectors (fig. 5*E*). The brain-expressed biogenic amine receptors include HTR4, TARL, and nonolfactory TAAR1, and the MOE-expressed olfactory amine detectors include TARLL and olfactory TAARs. The brain-expressed biogenic amine receptors mainly recognize ligands with aromatic rings (chemical cluster 3) that are known as amine neurotransmitters or trace amines, whereas the MOE-expressed olfactory amine detectors mainly recognize ligands that are primary and secondary alkyl amines (chemical cluster 2) as well as tertiary amines (chemical cluster 5). Our phylogenetic analysis suggests that the MOE-expressed TAARs likely evolved from the brain-expressed biogenic amine receptors. Similar evolutionary trajectories are observed in other olfactory receptor families, such as formyl peptide receptors and membrane-spanning 4A (MS4A), which initially function as receptors in the immune system (Greer et al. 2016; Dietschi et al. 2017).

### Evolution of Amine Recognition Motifs

Prior to this study, the known amine recognition sites in TAARs include Asp^3.32^ which is conserved in the majority of aminergic receptors, as well the noncanonical Asp^5.42^. Binding of monoamines requires either one of the two sites, whereas binding of diamines needs both sites (Li et al. 2015). With more receptors deorphaned in our study, we found several exceptions to this model and described receptors with distinct amine recognition motifs, further expanding our understanding of ligand-binding mechanisms in this family.

Firstly, we show that Asp^3.32^ is maintained in all TAARs and TARLs, suggesting the evolutionarily conserved monoamine recognition motif in amine-detecting receptors. Interestingly, the lamprey TARLL2 subfamily members also contain Asp^5.42^ (supplementary fig. S6*A*, Supplementary Material online). One member of TARLL2, TARLL2a (also called TAAR346a) has been deorphaned and shown to recognize cadaverine and spermine (Scott et al. 2019). It is very likely that both Asp^3.32^ and Asp^5.42^ in TARLL2a contribute to diamine or polyamine recognition, in a similar way to TAARs.

Secondly, exceptions to the use of canonical amine recognition motifs exist in TARLLs. Our homology modeling data suggest that binding of indole in TARLL1b requires Phe^5.38^ instead of Asp^3.32^ (fig. 4*F*). The existence of other binding sites besides Asp^3.32^ or Asp^5.42^ provides a possible structural basis for a few TAARs lacking either Asp^3.32^ or Asp^5.42^ (supplementary fig. S6*C*, Supplementary Material online). Surprisingly, we also found that TARLL3h without Asp^5.42^ is able to recognize diamines. TARLL3h possibly detects diamines through Asp^3.32^ and Tyr^3.33^, representing a novel diamine-binding mechanism.

Thirdly, we identified some interesting features of amine recognition motifs in TAARs. In clade II TAARs, one amino acid at 5.44 site is lost during evolution (supplementary fig. S6*A*, Supplementary Material online). Therefore, TAAR6 and TAAR8 subfamily members have Asp^5.43^ instead of Asp^5.42^. Previous studies have conjectured that these mammalian TAAR6 and TAAR8 can recognize diamines by computational analysis (Izquierdo et al. 2018). Here, we found that several TAAR6 and TAAR8 subfamily members can only detect monoamines but not diamines. Those results suggest that Asp^5.43^ does not function similarly to Asp^5.42^, possibly due to orientation of Asp^5.43^ away from the binding pocket. In addition, the TAAR9 subfamily members have Asp^3.32^ but not Asp^5.42^, yet mouse TAAR9 can recognize polyamines, including spermine, spermidine, and diamine such as cadaverine (Saraiva et al. 2016). Our experiments also detected activation of TAAR9 from rat, cat, rabbit, and hamster by spermidine, spermine, and cadaverine (supplementary table S3, Supplementary Material online), suggesting another diamine/polyamine recognition mechanism without Asp^5.42^ (Jia et al. 2021). Together, the evolutionary conservation and diversification of amine recognition motifs highlights the structural versatility of those amine-detecting receptors.

## Materials and Methods

### Phylogenic Tree Construction

The receptor sequences of human, mouse, rat, and zebrafish were retrieved in previous publication (Hussain et al. 2009; Saraiva et al. 2015). Among the identified species, zebrafish has the largest number of *Taar* genes. However, the exact number of zebrafish *Taar* genes is still controversal. Hussain et al. (2009) reported 112 *Taar* genes in zebrafish, whereas RNA-seq analysis of the zebrafish MOE samples revealed 118 *Taar* genes in another study (Saraiva et al. 2015). To acquire a more accurate collection of the zebrafish *Taar* gene repertoire, we validated the sequences from the two studies by comparing them with sequences in the Ensembl database. After deleting the pseudogenes and duplicated sequences, we eventually acquired 108 functional *Taar* genes (supplementary table S5, Supplementary Material online). The receptor sequences of other species were obtained using TBlastN against genome assemblies in NCBI. Sequences with abnormal characters that indicate inaccurate sequences were deleted unless we confirmed the correct sequences by cloning. The criteria used to determine a functional receptor include: 1) the coding sequence length longer than 750 bp; 2) the presence of seven transmembrane domains predicted by TMpred web server (https://embnet.vital-it.ch/software/TMPRED_form.html; last accessed January 13, 2022); 3) position within the TARLL, TARL, or TAAR clade in the constructed phylogenetic tree (see below). The sequences of functional receptors were included in the supplementary data S1, Supplementary Material online. For TAAR pseudogene identification, the numbers of pseudogenes of human, mouse, rat, horse, bat, elephant, armadillo, platypus, chicken, and zebrafish were obtained from a previous publication (Eyun et al. 2016). The pseudogenes of alligator, coelacanth, spotted gar, whale shark, and sea lamprey were retrieved by using the protein sequences of the functional TAARs/TARLs as a query in TBlastN against the genome of the corresponding species. The previously identified functional receptors and the new receptors with lengths less than 250 amino acids were removed. For the remaining receptors, additional BlastP searches were performed against human, mouse, rat, and zebrafish protein databases, and the receptors were retained as potential TAAR/TARL pseudogenes only if the most homologous aligned proteins were TAARs. Pseudogenes were defined as receptors containing interrupting stop codons, frameshifts, or deletions in conserved regions. The identified pseudogenes were listed in supplementary data S2, Supplementary Material online.

Multiple sequence alignment was carried out by MAFFT v7.313 using E-INS-I option and an opening gap penalty of 1.8, intending to reduce gaps as many as possible (Katoh and Standley 2013). TrimAl was applied to trim columns with gaps at a percentage over 90% (Capella-Gutierrez et al. 2009). The phylogenetic tree was constructed by ML. The amino acid substitution model (JTT+G4) was automatically selected by ModelFinder (Kalyaanamoorthy et al. 2017) in Phylosuite (Zhang et al. 2020). ML trees were built using IQ-TREE v1.6.8 (Nguyen et al. 2015) integrated in Phylosuite; multiple runs produced similar results. Nodal support was assessed both by 5,000 replicates of the UF bootstrap approximation (Hoang et al. 2018) implemented in IQ-TREE, as well as 100 standard bootstrap replicates. Figtree v1.4.4 was used to visualize and modify the phylogenetic tree (http://tree.bio.ed.ac.uk/software/figtree/; last accessed January 13, 2022). The species tree was generated using Timetree (http://www.timetree.org; last accessed January 13, 2022) and visualized in Figtree v1.4.4. Sequence logo figures were generated by WebLogo (Crooks et al. 2004). Transmembrane regions were determined according to human TAAR9 predicted by GPCRdb (Pandy-Szekeres et al. 2018).

### Chemical Clustering Analysis

The structures of 97 small molecules were retrieved from PubChem (https://pubchem.ncbi.nlm.nih.gov; last accessed January 13, 2022). Hierarchical clustering of these ligands was generated in the Canvas module integrated in Schrödinger Suite platform for chemical analysis (Sastry et al. 2010). We appointed MACCS (Molecular ACCess System) binary fingerprints for each molecule and ran the hierarchical clustering (Durant et al. 2002). The results were displayed using the Kelley criterion (Kelley et al. 1996) to calculate distances and generate clusters.

### Homology Modeling and Ligand Docking

Homology models of sea lamprey TARLL1b and TARLL3h were generated using GPCR-I-TASSER (Zhang et al. 2015). The predicted models were refined to the prepared states to adopt a physiological state by Protein Preparation Wizard module integrated in Schrödinger Suite (Sastry et al. 2013). The ligands were prepared by LigPrep of Maestro (Maestro 2020), with the states consistent with physiological pH. We then performed receptor–ligand docking in the Induced-Fit Docking module of the Schrödinger platform by generating several poses of ligand–receptor interactions (Farid et al. 2006). The final poses were selected according to the docking score and glide model.

### Chemicals

Amine compounds were purchased from Sigma–Aldrich. Nonamine compounds were kindly donated by Hanyi Zhuang’s lab (Shanghai Jiao Tong University School of Medicine). All chemicals were dissolved in distilled water, DMSO or anhydrous ethanol at the concentration of 10 mM and stored at −20 °C. The detailed information of all chemicals was included in supplementary table S6, Supplementary Material online.

### Cell Lines

HEK293T cells used for the SEAP assay were cultured in Dulbecco’s-modified eagle medium (DMEM, Thermo Fisher Scientific) supplemented with 10% bovine calf serum (BCS, HyClone) and 5% penicillin–streptomycin solution (Thermo Fisher Scientific). The HEK293T-derived Hana3A cell line used for Dual-Glo luciferase assay was grown in DMEM with 10% BCS, 5% penicillin–streptomycin solution with or without 1 μg/ml puromycin (ApexBio). Both cell lines were cultured at 37 °C with 5% CO_2_.

### Genomic DNA Used for Receptor Cloning

All receptor genes were cloned from genomic DNA except zebrafish *Htr4* that was cloned from zebrafish brain cDNA (AB strain). Sea lamprey genomic DNA was provided by Dr Jeramiah Smith from University of Kentucky. Fugu genomic DNA was provided by Dr Byrappa Venkatesh from National University of Singapore. Pufferfish genomic DNA was provided by Dr Ferenc Mueller from University of Birmingham. Medaka genomic DNA was provided by Dr Ivan Conte from the Telethon Institute of Genetics and Medicine (TIGEM). Coelacanth genomic DNA was provided by Dr Jeremy Johnson from the Broad Institute. Brownbanded bamboo shark and spotted gar were purchased from commercial suppliers, sacrificed following protocols approved by the Harvard University Animal Care and Use committee (IACUC); tissue samples are accessioned in the Museum of Comparative Zoology (Ichthyology: bamboo shark No. 171795, spotted gar No. 171800). Genomic DNA of catfish, salmon, alligator, chicken, dog, rabbit, cow, guinea pig, hamster, cat, horse, sheep, wild boar, and rhesus were purchased from Zyagene. The target fragments were inserted into pcDNA3.1—(Invitrogen) or modified pcDNA3.1—that contains an addition of DNA fragments encoding the N-terminal 20 amino acids of bovine rhodopsin in the N-terminal of target fragments.

### SEAP Assay

HEK293 cells were plated onto poly-d-lysine (Sigma–Aldrich) preincubated 96-well plates (Corning) with 50 µl growth medium (DMEM medium with 10% BCS and 5% penicillin–streptomycin solution) and cultured for 18–24 h. Receptor plasmids were cotransfected with Cre-SEAP plasmid using polyethylenimine (PEI, Polysciences) and incubated at 37 °C with 5% CO_2_. About 4 h later, cells were incubated with or without test compounds diluted in serum-free DMEM for 48 h, followed by incubating for 2 h at 70 °C. After returning to room temperature, 50 µl supernatant from each well was incubated with an equal volume of 0.3 mM 4-methylumbelliferyl phosphate (4-MUP, Sigma–Aldrich) in 2 M diethanolamine bicarbonate (Sigma–Aldrich), pH 10.0 for 15 min. Fluorescence was measured with a BioTek Microplate reader.

### Dual-Glo Luciferase Assay

Hana3A cells were plated onto 96-well plates (Greiner bio-one) with 50 µl growth medium (DMEM medium with 10% BCS and 5% penicillin–streptomycin solution with puromycin) and cultured at 37 °C with 5% CO_2_. After 18–24 h, cells in each well were cotransfected with 50 ng receptor plasmid, 10 ng CRE-Luc, 10 ng pRL-SV40, and 10 ng mRTPs using Lipofectamine 2000 (Invitrogen), and incubated for 18 h. Medium was then replaced by 25 µl CD293 (Invitrogen) containing 1% glutamine with or without test compounds for 4 h. The chemiluminescence of firefly luciferase and renilla luciferase were measured with a Biotek Microplate reader using the Dual-Glo Luciferase Assay System (Promega).

### Analyses of Functional Assays

We conducted phylogenetic principle component analyses (phyloPCA) on the receptor response data (either binary responses of all deorphaned receptors or continuous values of HTR4, TARL, TAARC1 and TAAR1 to 16 ligands) together with the trimmed ML trees defined above using phly.pca() in the phytools package of R (Revell 2009). For the phyloPCA output of all receptor responses, we applied UMAP on the scores of all 52 PCs to further reduce dimensions and visualize on two UMAP axes. We also used fviz_nbclust() in R package factoextra (Kassambara and Mundt 2020) to determine the optimal number of clusters of deorphaned receptors using all phyloPCA scores. When setting the maximum number of clusters to be less than 12, the optimal number of clusters was two. We then used PAM (Partitioning Around Medoids) to identify which receptors belonged to each of the two clusters in the R package cluster (Maechler et al. 2019). To detect past shifts in functional profiles across the phylogeny, we applied l1ou to scores on the first five PCs (which explained 58.2% of the total variance) from the all-receptor phyloPCA output using the pBIC criterion (Khabbazian et al. 2016). The l1ou approach can efficiently use multiple traits to compute the number of shifts across the phylogeny under an Ornstein–Uhlenbeck process using a LASSO (Least Absolute Shrinkage and Selection Operator) procedure (Tibshirani 1996) without any prior hypotheses regarding locations of shifts. To compare the effect of the slightly different topology reported in Dieris et al. 2021, we trimmed both topologies to include the shared taxa (34 receptors with identified ligands) and performed phyloPCA and clustering analyses as described above. l1OU analyses were performed using the scores of all PCs on both reduced data sets.

### In Situ Hybridization

Sea lamprey *Tarll* antisense probes were designed against the entire coding region of each receptor gene. The primers used for making probes were included in supplementary table S7, Supplementary Material online. Coding region fragments were amplified from TARLL plasmids, and used for synthesis of digoxigenin-labeled RNA probes with DIG RNA labeling kit (SP6/T7). Sea lamprey MOE sections were cut at 14 µm, and hybridized with RNA probes with 1:200 dilution in the hybridization solution at 58 °C overnight. On the second day, sections were washed in 5× SSC (Invitrogen) two times for 5 min and 0.2× SSC two times for 30 min at 70 °C. The sections were incubated with blocking buffer at room temperature for 1 h, and then incubated with alkaline phosphatase-conjugated peroxidase-anti-DIG (Roche) with 1:500 dilution in the blocking buffer for 1 h at room temperature. Finally, sections were incubated in nitro blue tetrazolium and 5-bromo-4-chloro-3-indolyl phosphate (NBT/BCIP, Thermo Fisher Scientific) with 1:50 dilution in the alkaline phosphatase buffer at room temperature until strong signals were observed. The procedures of sea lamprey handling were approved by the Michigan State University Institutional Animal Use and Care Committee (03/14-054-00 and 02/17-031-00).

### Quantitative PCR (qPCR)

Zebrafish RNA of five tissues (brain, MOE, heart, kidney, and liver) were extracted from zebrafish (AB strain) with Trizol (Invitrogen), and reverse transcribed into cDNA with RT SuperMix (ApexBio). qPCR was performed using 10 µl reaction system containing SYBR green indicator (Roche), cDNA from each tissue, qPCR primers for receptor genes. The qPCR primers were included in supplementary table S7, Supplementary Material online. Handling procedures of zebrafish were approved by the Institute of Neuroscience, Chinese Academy of Sciences.

## Supplementary Material

Supplementary data are available at *Molecular Biology and Evolution* online.

## Supplementary Material

msac006_Supplementary_Data
