# Correction to: Evolution of Brain-Expressed Biogenic Amine Receptors into Olfactory Trace Amine-Associated Receptors

**DOI:** 10.1093/molbev/msac072

**Published:** 2022-05-30

This is a correction to: Lingna Guo, Wenxuan Dai, Zhengrong Xu, Qiaoyi Liang, Eliot T Miller, Shengju Li, Xia Gao, Maude W Baldwin, Renjie Chai, Qian Li, Evolution of Brain-Expressed Biogenic Amine Receptors into Olfactory Trace Amine-Associated Receptors, Molecular Biology and Evolution, Volume 39, Issue 3, March 2022, msac006, https://doi.org/10.1093/molbev/msac006

In the originally published version of this manuscript, the second affiliation and the Acknowledgement section were incorrect.

The second affiliation was originally published as:

2 Department of Anatomy and Physiology, Key Laboratory of Cell Differentiation and Apoptosis of Chinese Ministry of Education, Shanghai Jiao Tong University School of Medicine, Shanghai, China.

This has been amended to:

2 Department of Anatomy and Physiology, Ministry of Education-Shanghai Key Laboratory of Children's Environmental Health in Xinhua Hospital, Shanghai Jiao Tong University School of Medicine, Shanghai, China.

In the Acknowledgement section, the following text:

“This work was supported by National Natural Science Foundation of China (31771154, 32122038, and 31970933 to Q.L., 82030029 and 81970882 to R.C.), the National Key Research and Development Program of China (2021ZD0203100)”,

has been updated as follows:

“This work was supported by the National Key Research and Development Program of China (2021ZD0203100), National Natural Science Foundation of China (31771154, 32122038, and 31970933 to Q.L., 82030029 and 81970882 to R.C.), Innovative research team of high-level local universities in Shanghai (SHSMU-ZDCX20211102),”.

