# Supplementary material for: Evolution of Brain-Expressed Biogenic Amine Receptors into Olfactory Trace Amine-Associated Receptors
Source: Mol Biol Evol. 2022 Jan 11;39(3):msac006. doi: 10.1093/molbev/msac006 (PMC8890504; doi:10.1093/molbev/msac006)
Supplement: msac006_Supplementary_Data [file msac006_supplementary_data.zip › figS5.pdf]

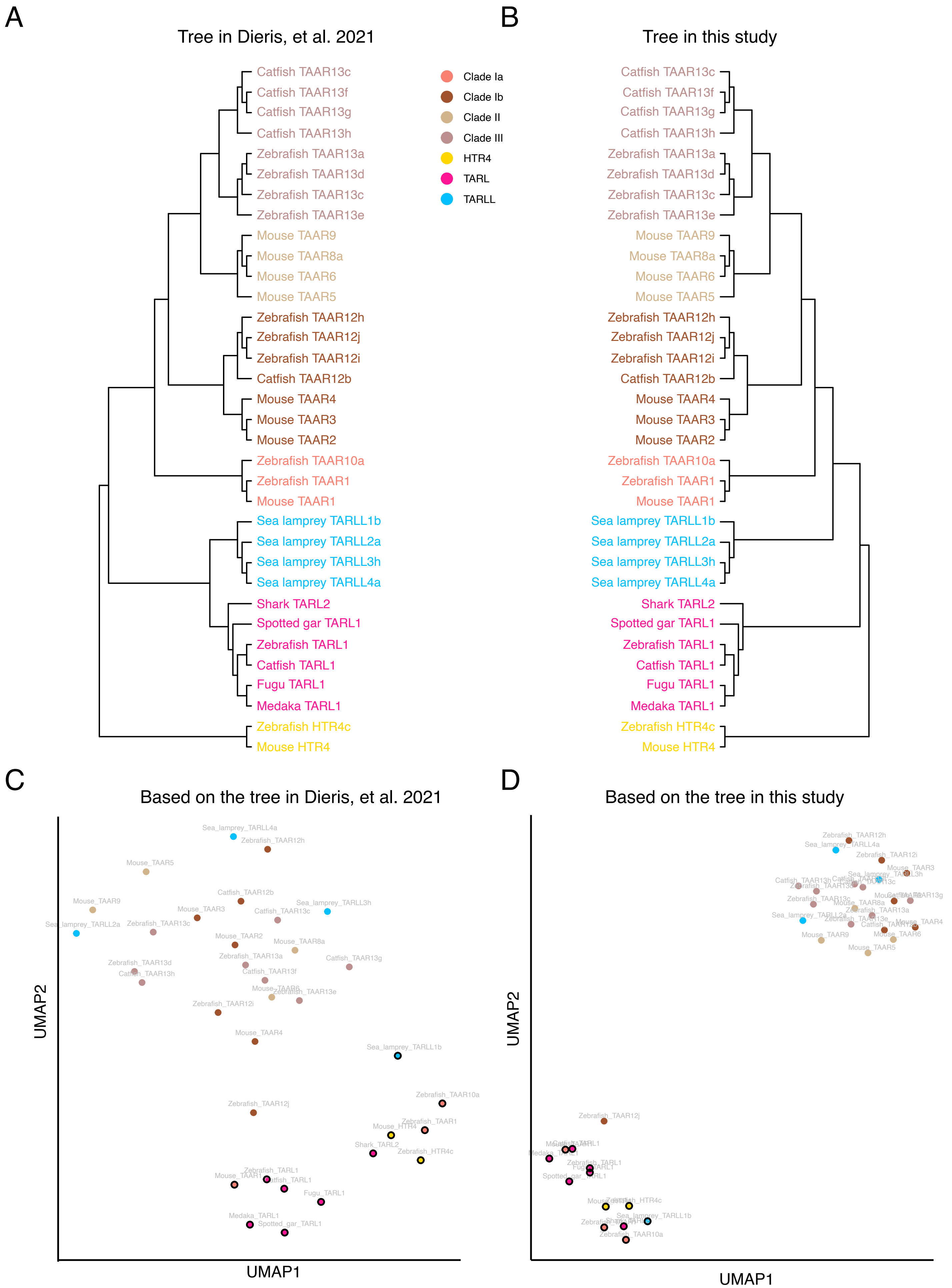

Supplementary figure 5
