# Supplementary material for: Evolution of Brain-Expressed Biogenic Amine Receptors into Olfactory Trace Amine-Associated Receptors
Source: Mol Biol Evol. 2022 Jan 11;39(3):msac006. doi: 10.1093/molbev/msac006 (PMC8890504; doi:10.1093/molbev/msac006)
Supplement: msac006_Supplementary_Data [file msac006_supplementary_data.zip › tableS7.pdf]

Supplementary table S7. List of primers for *in situ* probe production and qPCR.

**Target**	**Forward primer sequence**	**Reverse primer sequence**	**Product size [bp]**
	***In situ* probe production**		
TARLL1b	ATGAACTCTAGCCTGGCTCT	TCACAGCTCGGCTGAGCGGC	1071
TARLL4a	ATGTCATCTCACGCCAACAC	TTATCCGCGGGCTTTAGCAA	1047
TARLL3i	ATGGAAAACCGCACCGTGGG	CTACTTCACCCCGGACAGGT	1059
	**qPCR**		
zTAAR1	TCTACATGGTCGCCCGAAAC	GTTGCCTTGCGTGTCTGAAG	106
zTAAR10a	TGTCATGCTCGGTGTGTATC	TTTCCCTCCTTCTTCAGTTCAG	96
zTAAR12h	GCAGGCAGTCATTTCCAAACC	TCACCGGGAGTTGAGAAGTTG	149
zTARL	GGGTACATTAACTCCTCGCTAAA	CTGAGCCATGCAGACAAATTC	109
zHtr4	CCGTCGTGCCTTCCTTATCA	AGAGGAGCAGCCGTTCAGTA	133
zGAPDH	CATCTTTGACGCTGGTGCTG	TGGGAGAATGGTCGCGTATC	179
