# Supplementary figures and images for: Evolution of Brain-Expressed Biogenic Amine Receptors into Olfactory Trace Amine-Associated Receptors
Source: Mol Biol Evol. 2022 Jan 11;39(3):msac006. doi: 10.1093/molbev/msac006 (PMC8890504; doi:10.1093/molbev/msac006)

A

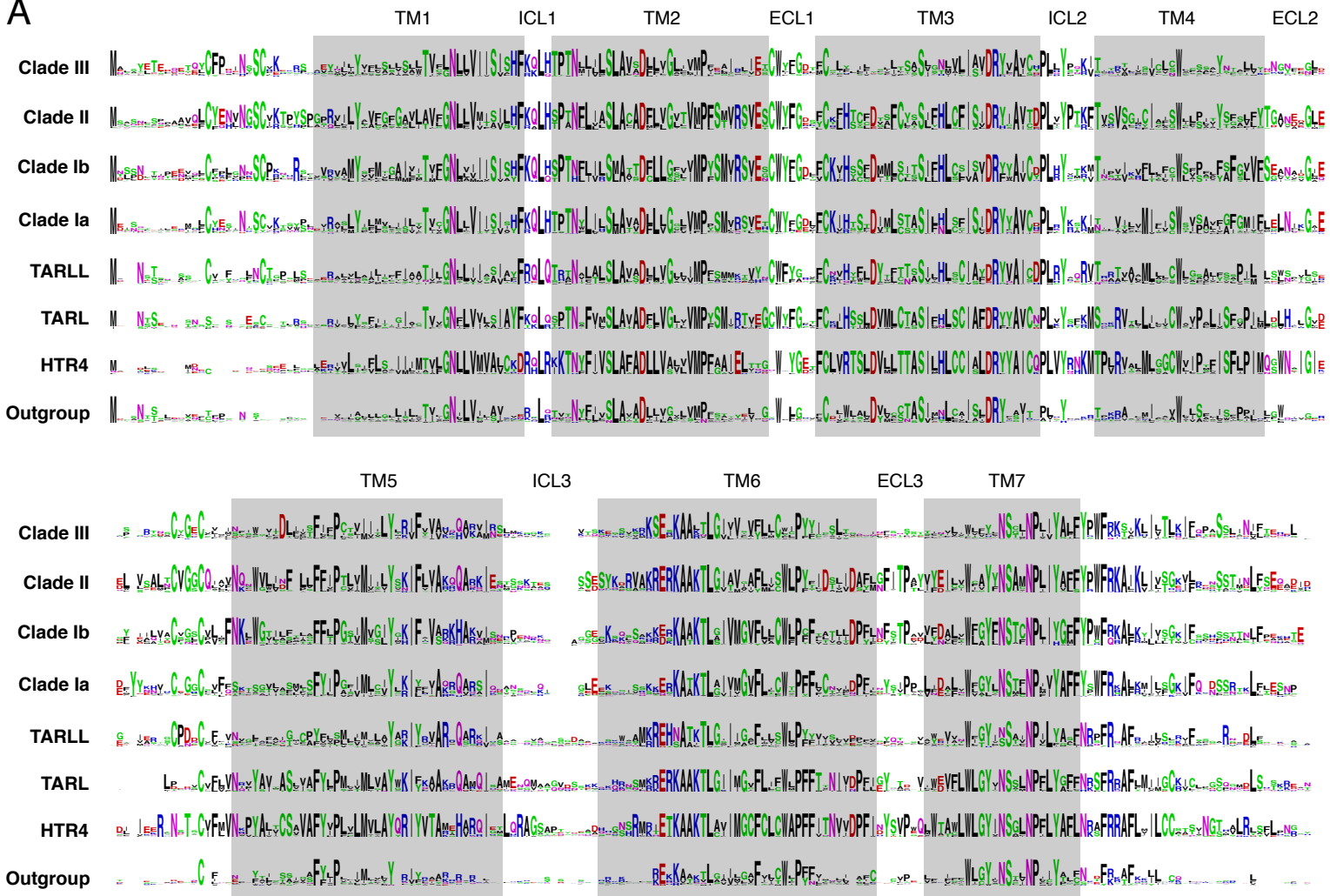

B

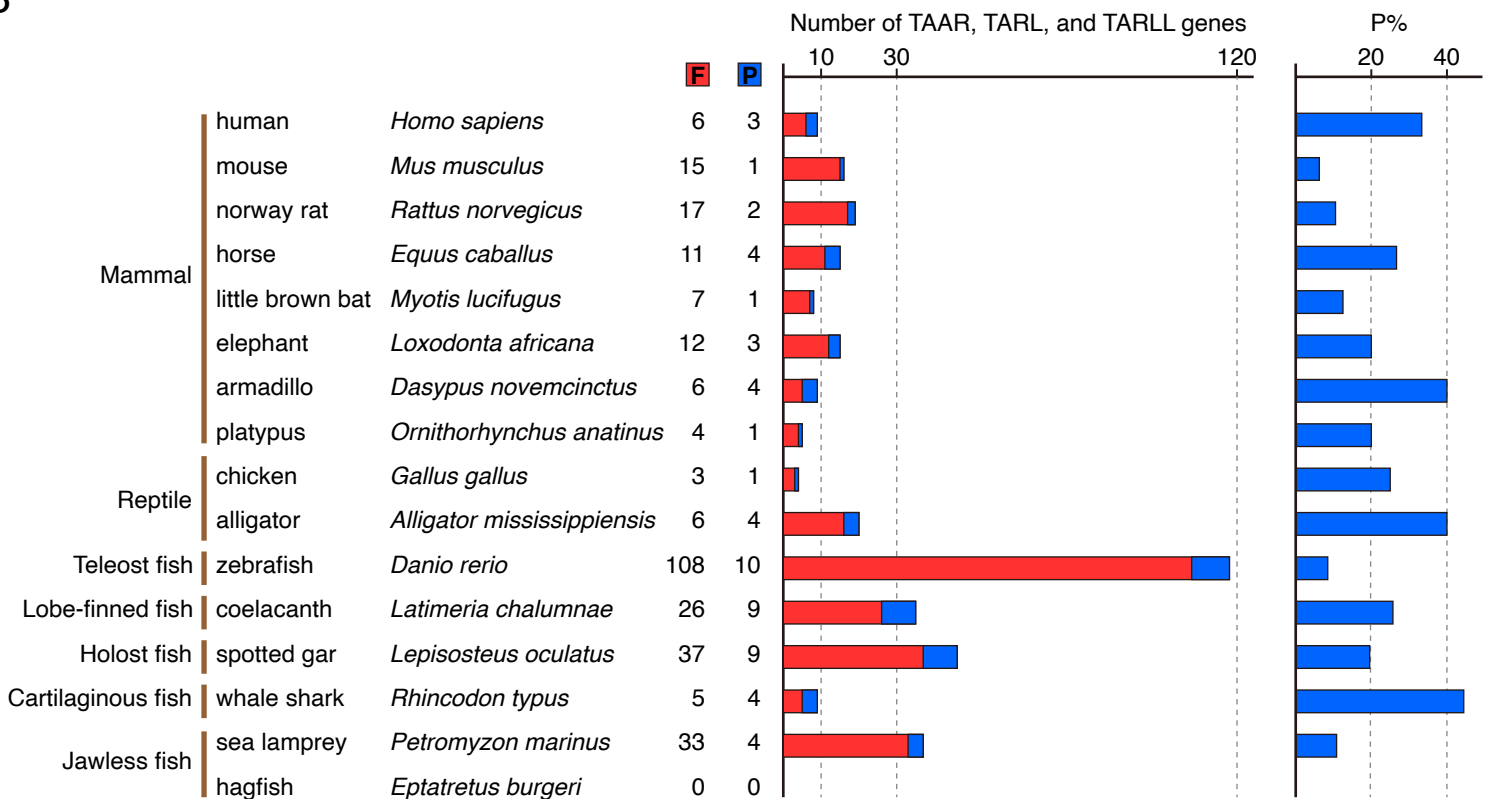

Supplementary figure 2

Supplement: msac006_Supplementary_Data [file msac006_supplementary_data.zip › figS2.pdf]

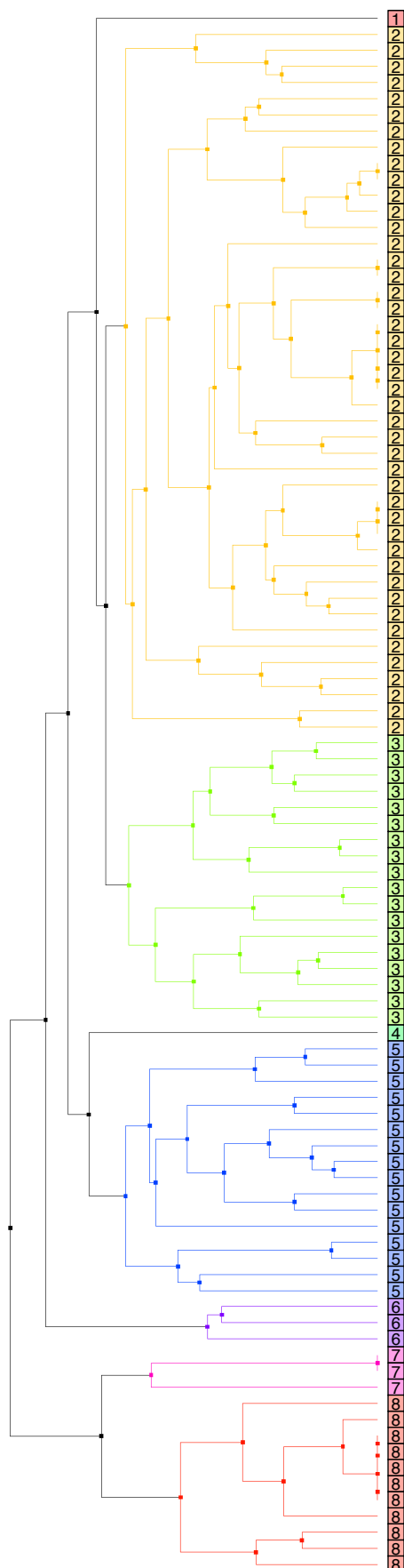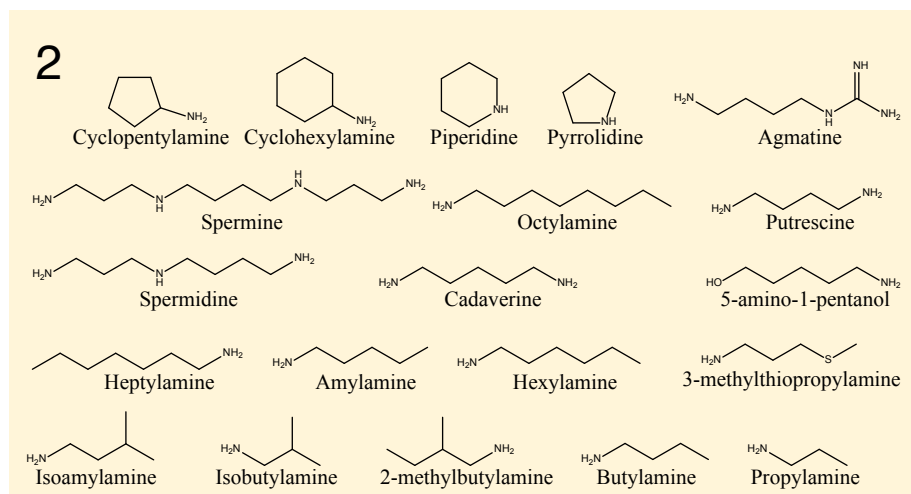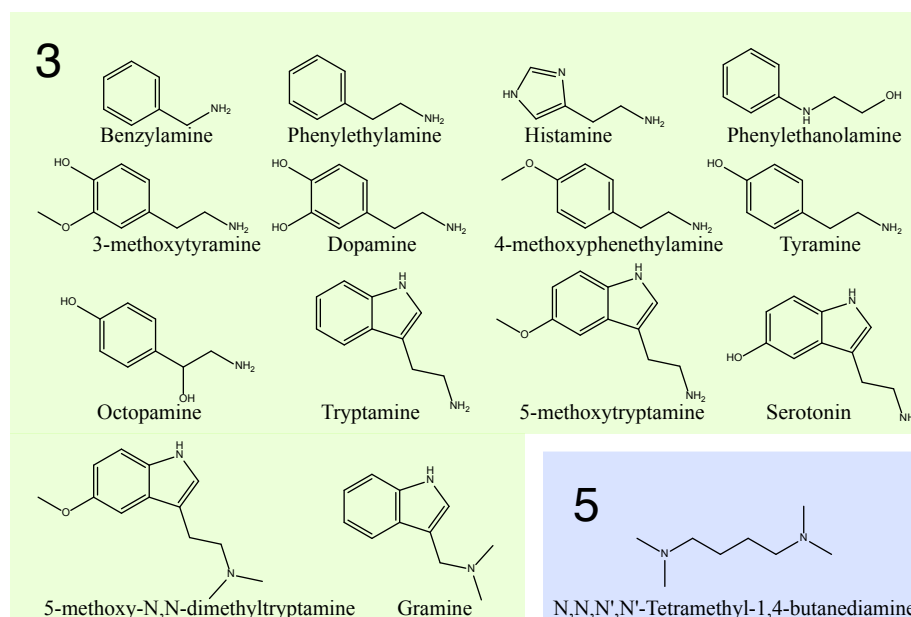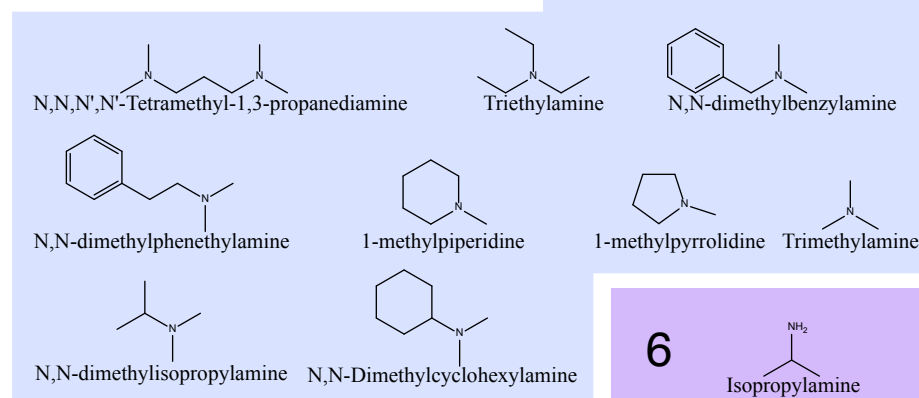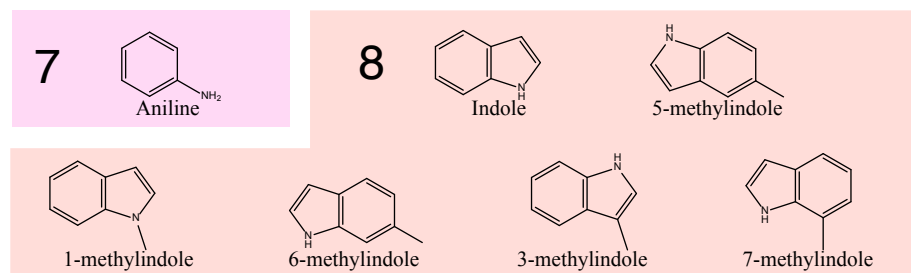

Supplementary figure 3

Supplement: msac006_Supplementary_Data [file msac006_supplementary_data.zip › figS3.pdf]

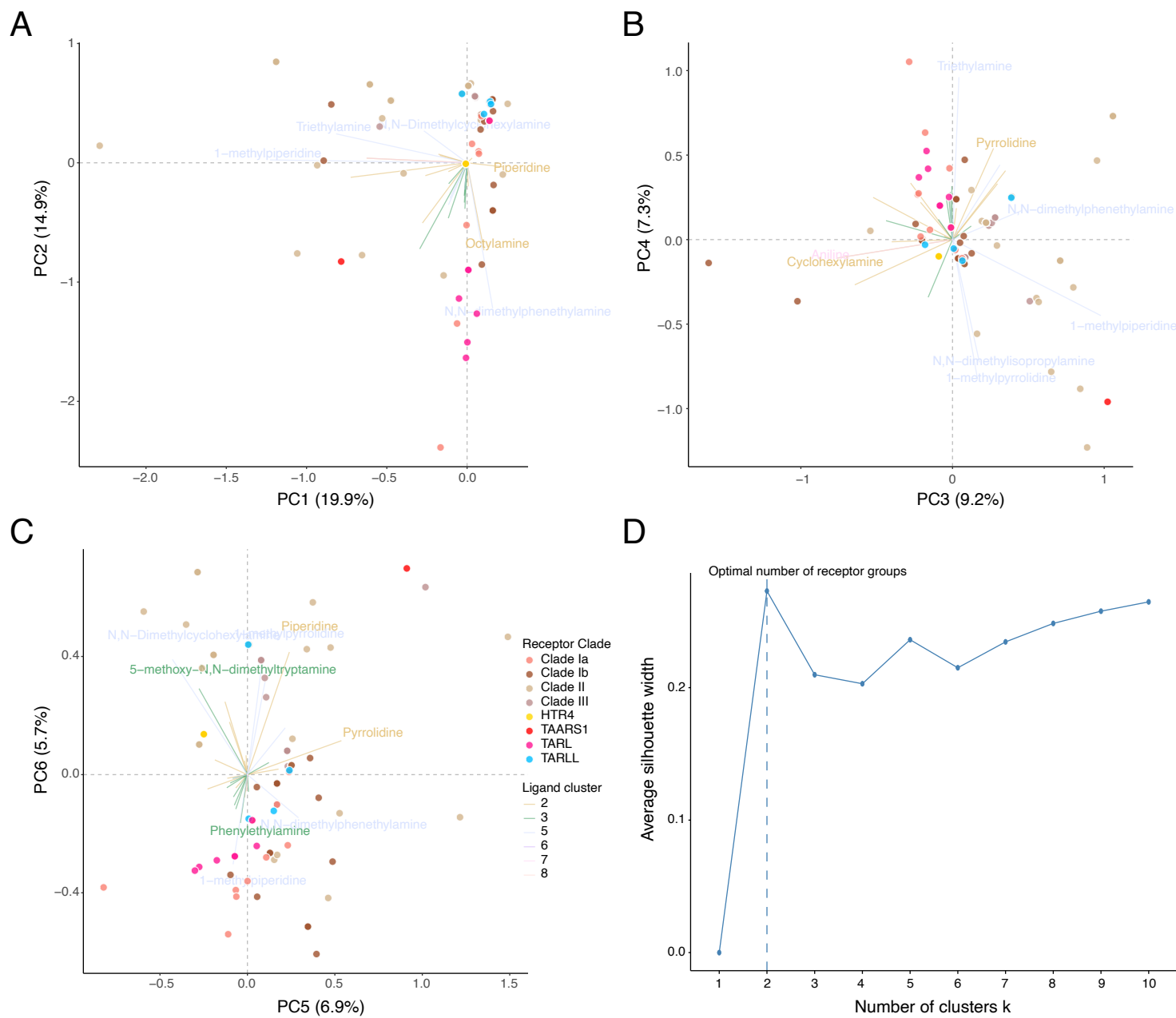

Supplementary figure 4

Supplement: msac006_Supplementary_Data [file msac006_supplementary_data.zip › figS4.pdf]

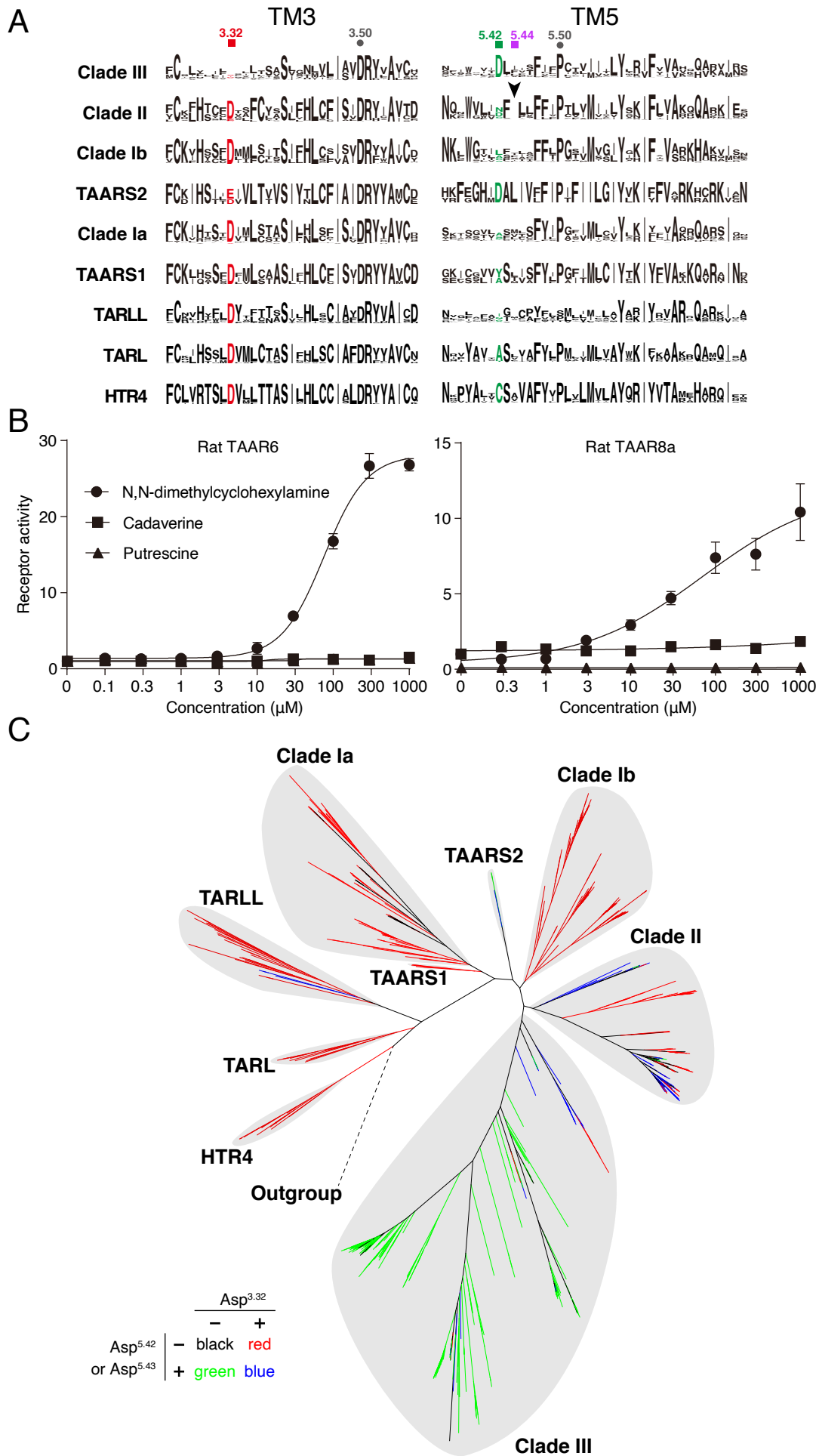

Supplementary figure 6

Supplement: msac006_Supplementary_Data [file msac006_supplementary_data.zip › figS6.pdf]

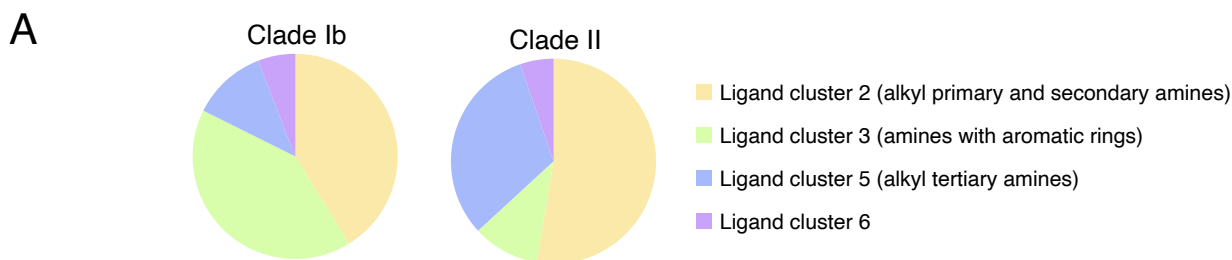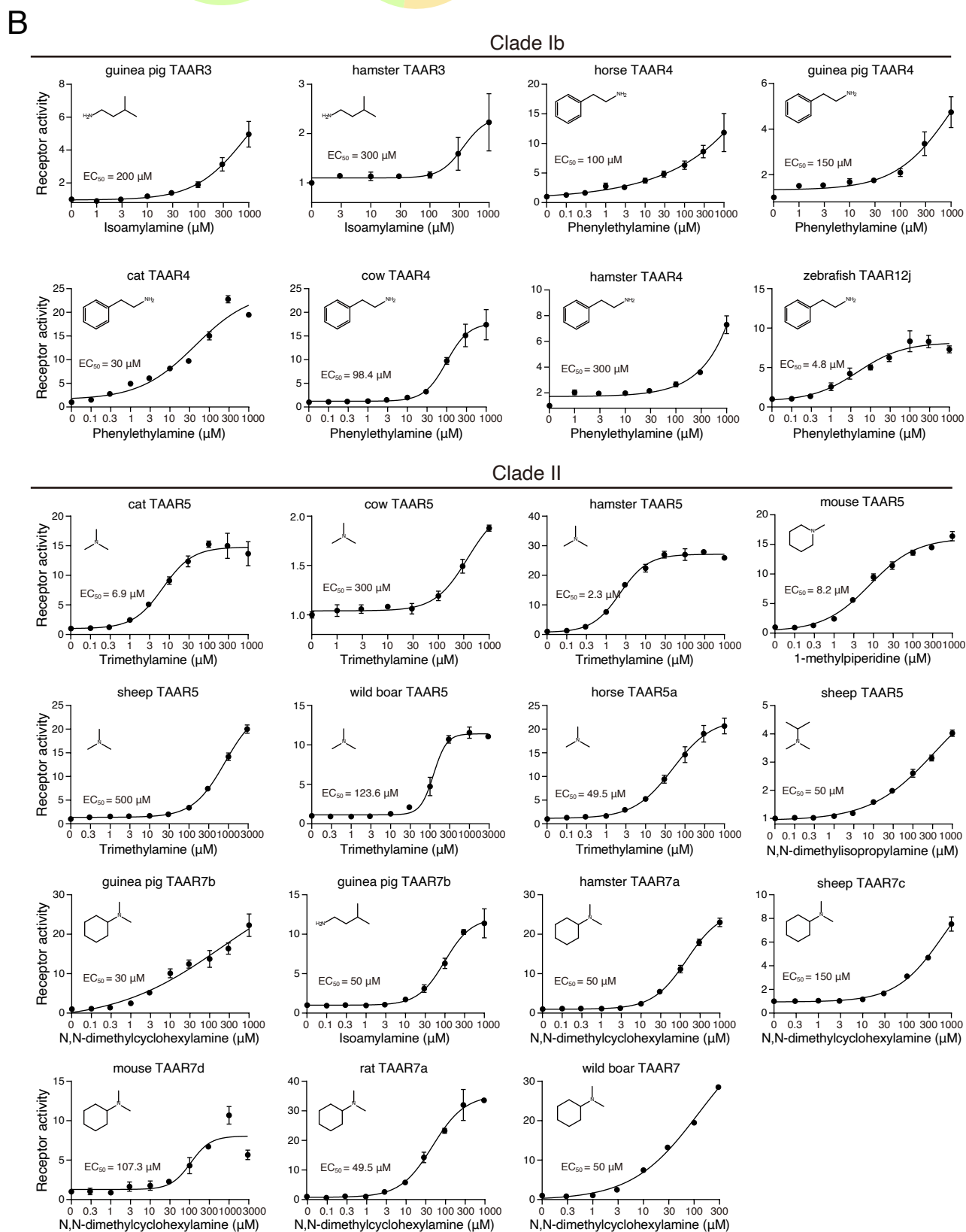

Supplementary figure 7

Supplement: msac006_Supplementary_Data [file msac006_supplementary_data.zip › figS7.pdf]

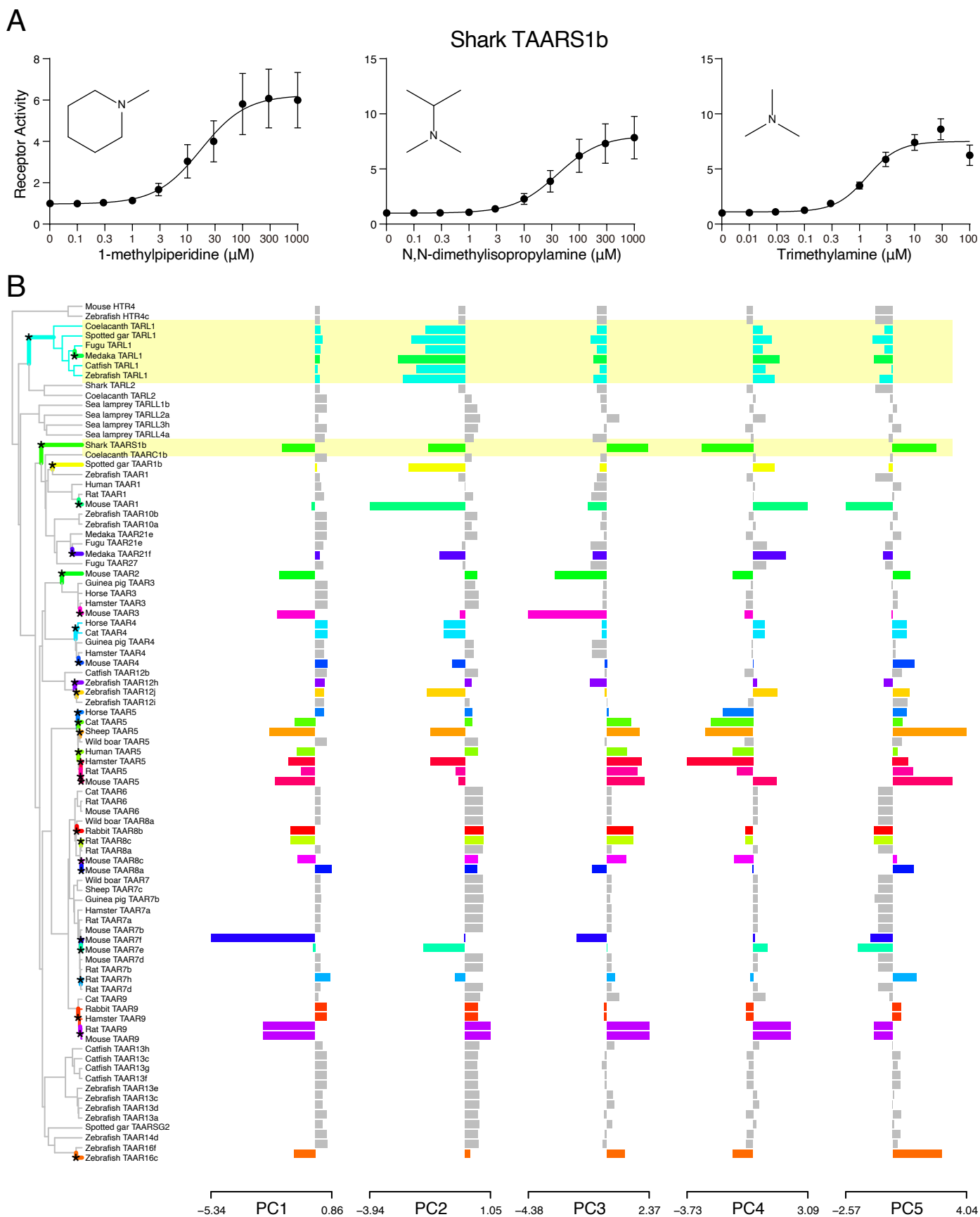

Supplementary figure 8

Supplement: msac006_Supplementary_Data [file msac006_supplementary_data.zip › figS8.pdf]

A

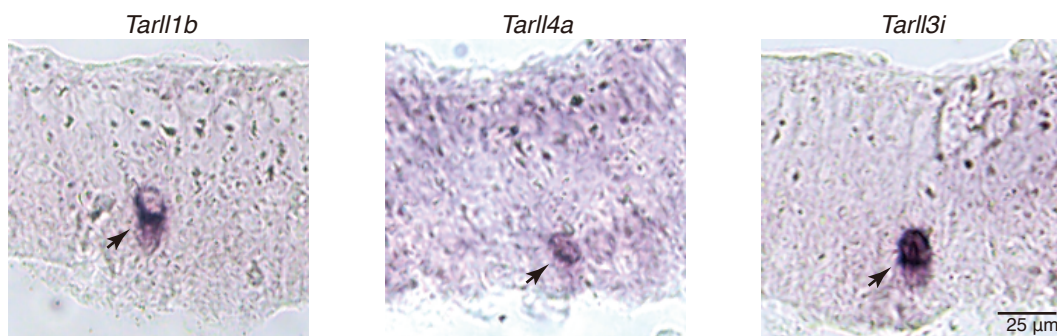

B

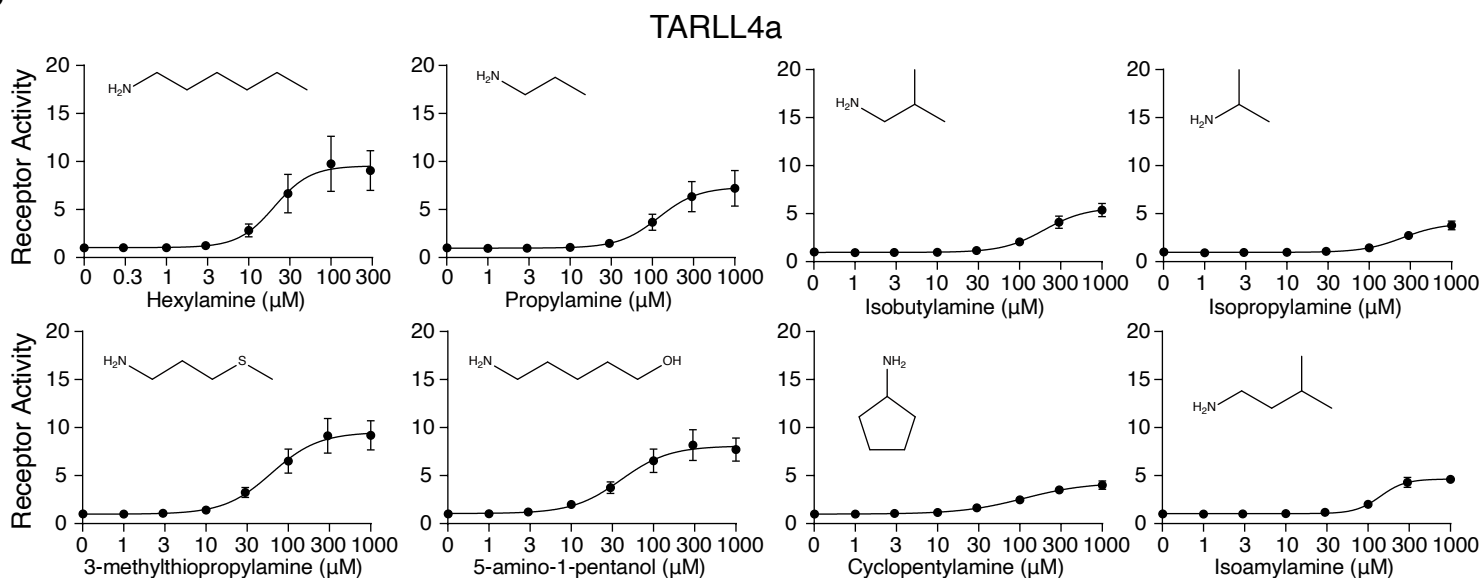

C

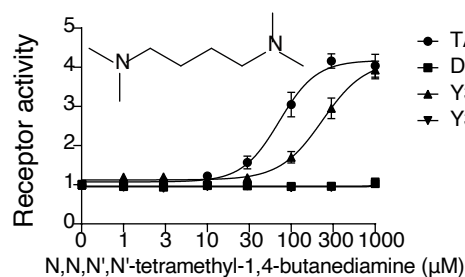

D

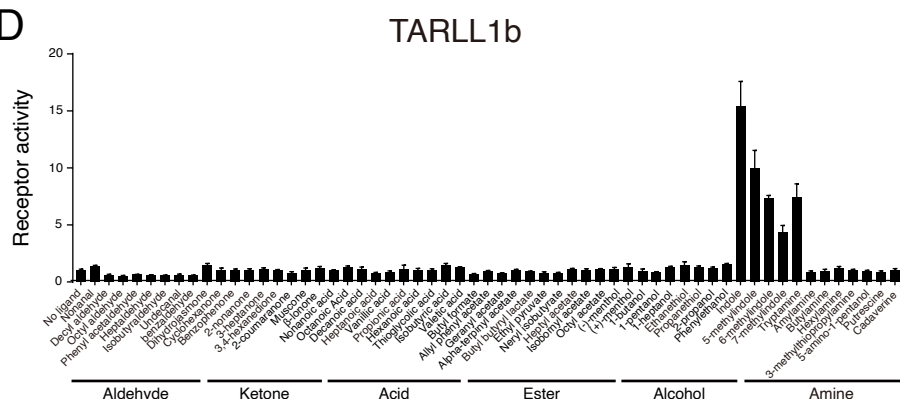

E

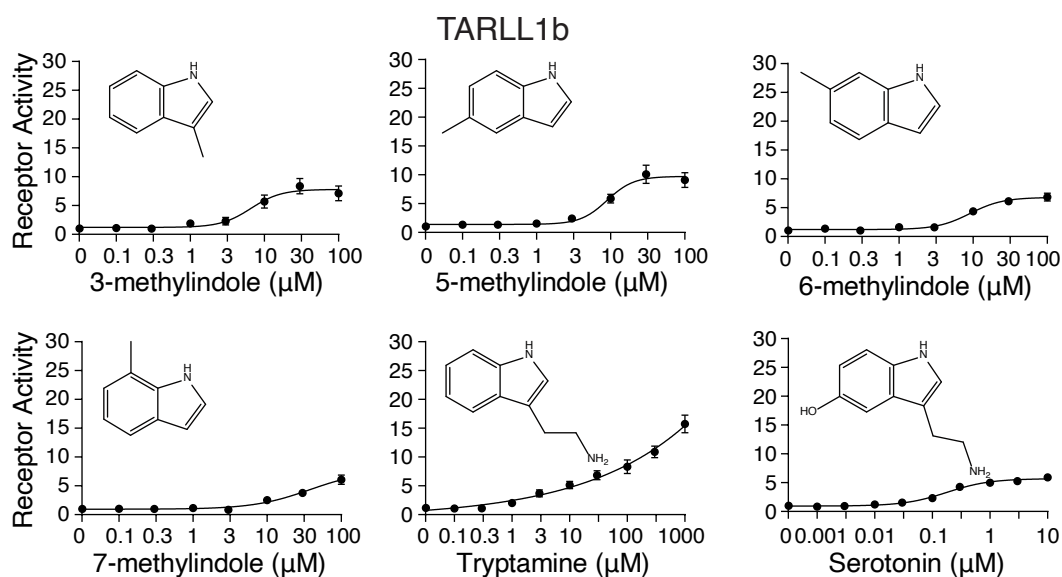

Supplementary figure 9

Supplement: msac006_Supplementary_Data [file msac006_supplementary_data.zip › figS9.pdf]

A

Based on the tree in Dieris, et al. 2021

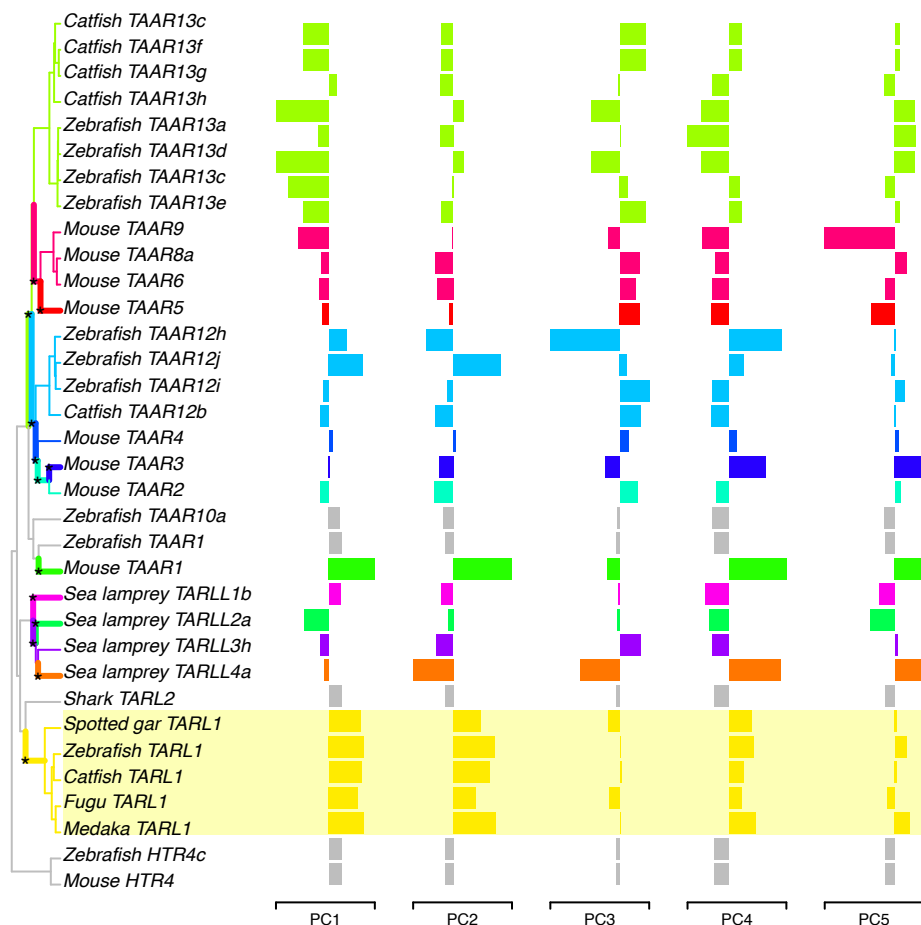

B

Based on the tree in this study

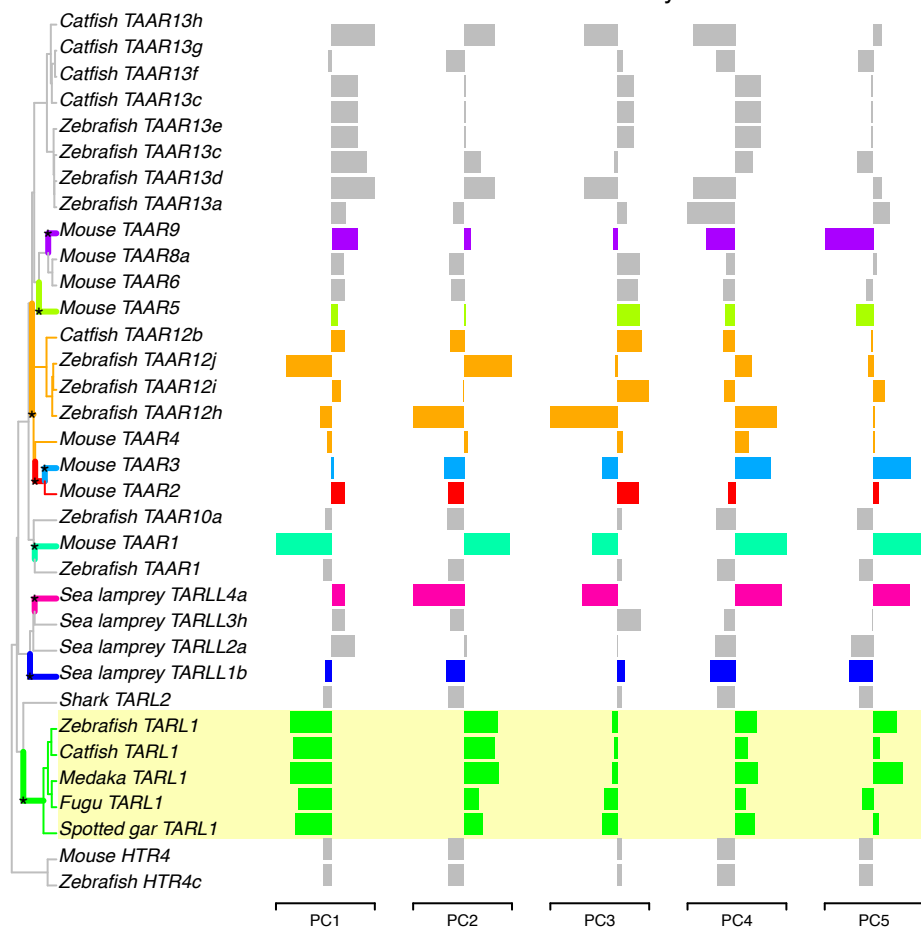

Supplementary figure 10

Supplement: msac006_Supplementary_Data [file msac006_supplementary_data.zip › figS10.pdf]

A

Tryptamine

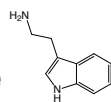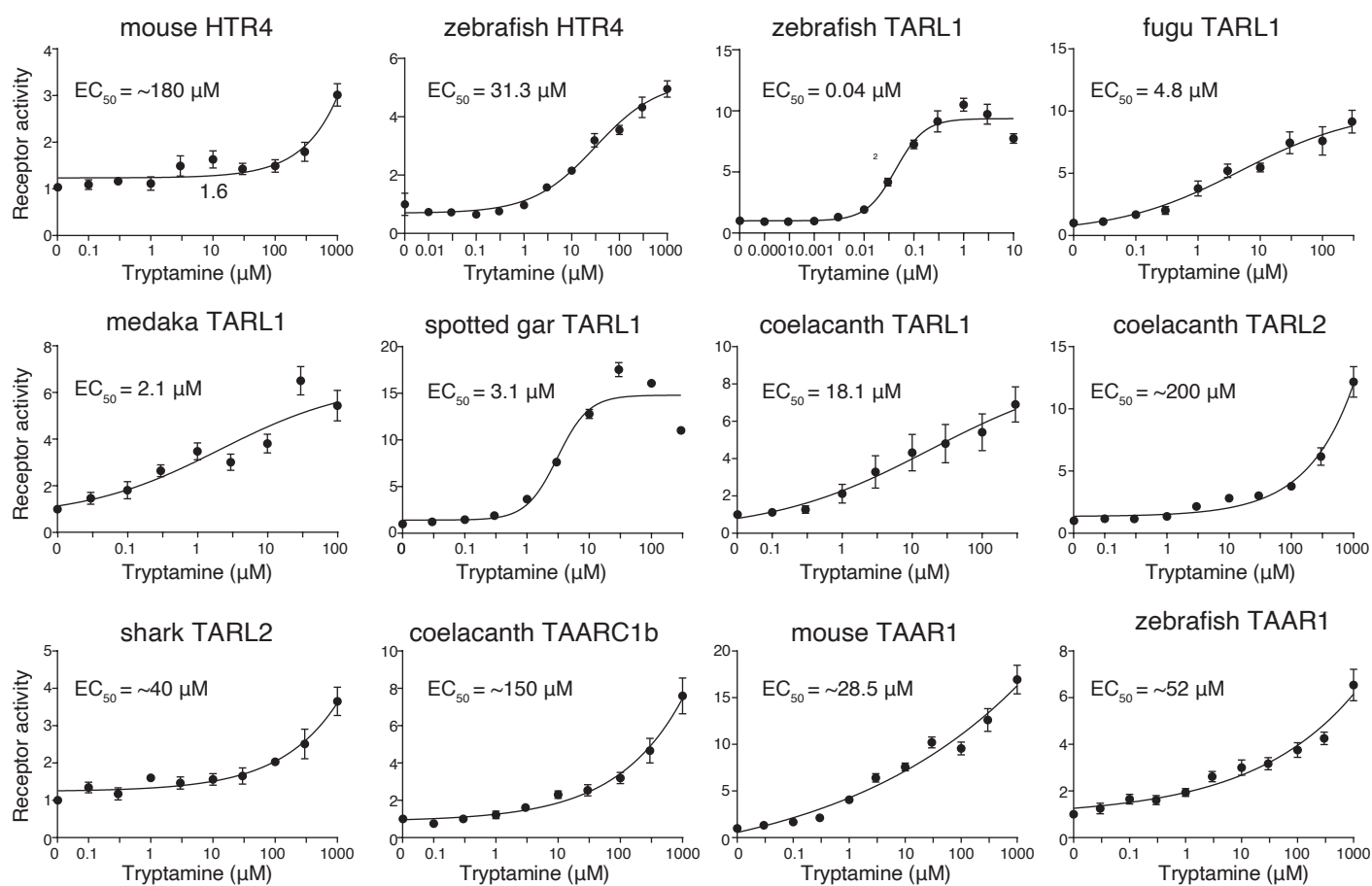

B

Serotonin

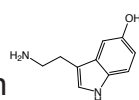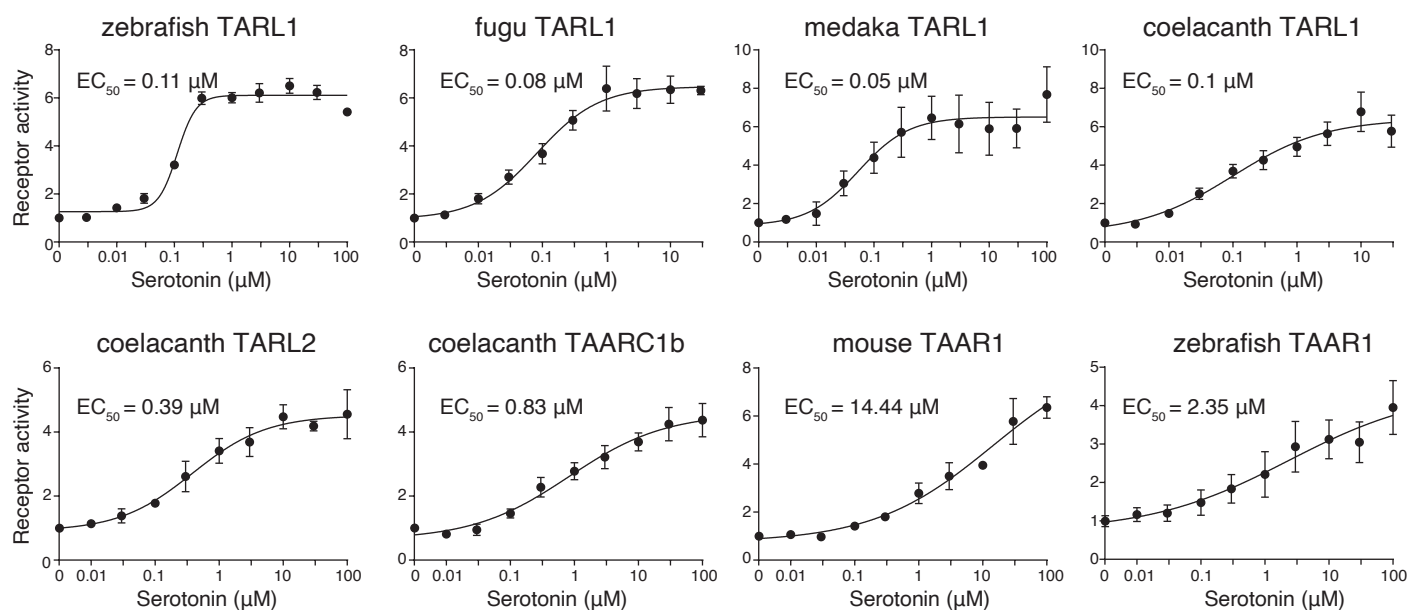

Supplementary figure 11

Supplement: msac006_Supplementary_Data [file msac006_supplementary_data.zip › figS11.pdf]

A

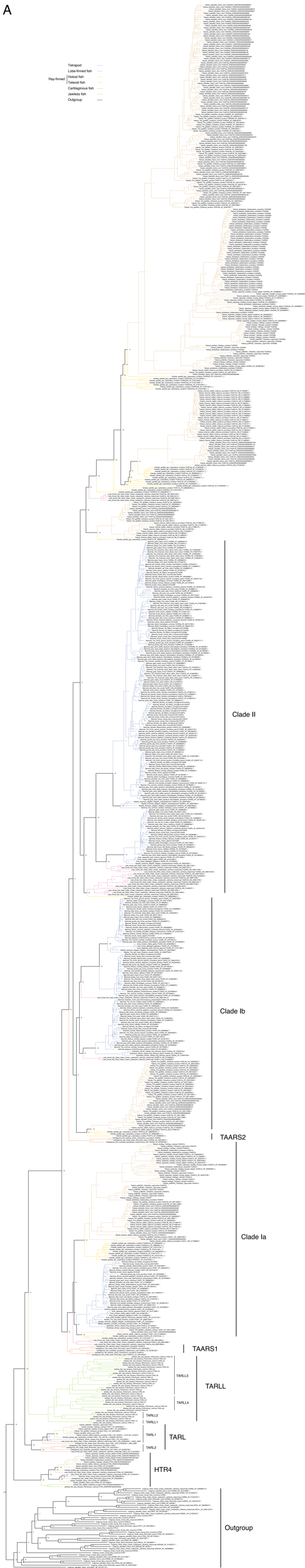

B

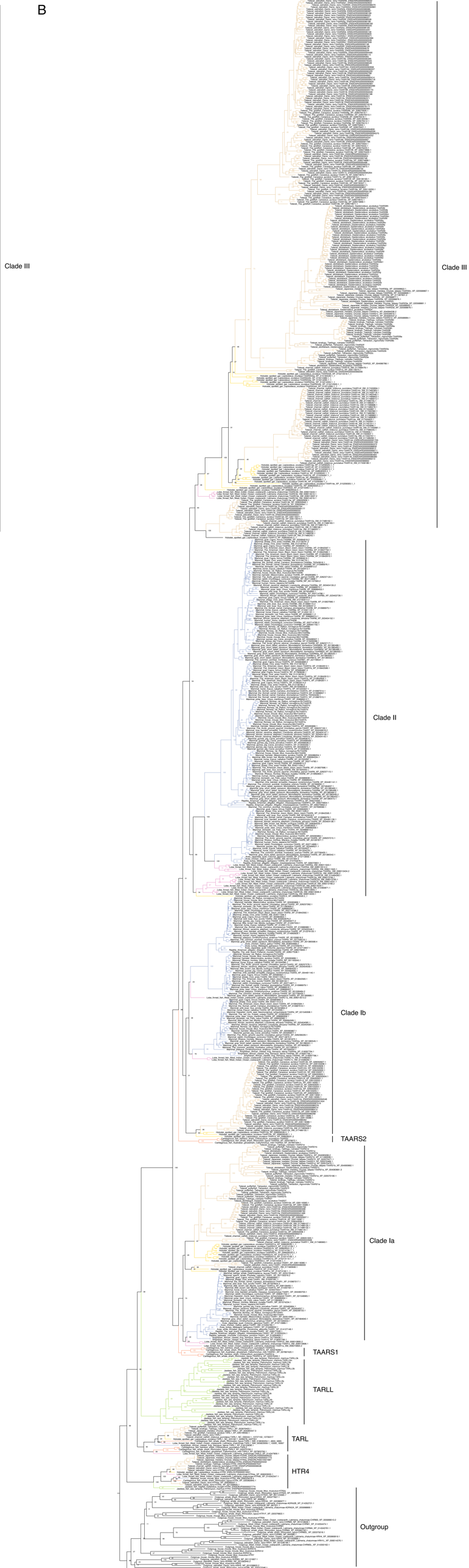

Supplementary figure 1

Supplement: msac006_Supplementary_Data [file msac006_supplementary_data.zip › figS1.pdf]
